# Molecular mapping and validation of quantitative trait loci for content of micronutrients in wheat grain

**DOI:** 10.3389/fpls.2024.1522465

**Published:** 2025-01-17

**Authors:** Xiangdong Chen, Junchao You, Nannan Dong, Di Wu, Die Zhao, Rui Yong, Wenjing Hu

**Affiliations:** ^1^ Henan Provincial Key Laboratory of Hybrid Wheat, School of Agriculture, Henan Institute of Science and Technology, Xinxiang, China; ^2^ Lixiahe Institute of Agriculture Sciences, Key Laboratory of Wheat Biology and Genetic Improvement for Low & Middle Yangtze Valley, Ministry of Agriculture and Rural Affairs, Yangzhou, Jiangsu, China

**Keywords:** wheat (*Triticum aestivum* L.), micronutrient, quantitative trait loci, mapping, breeding

## Abstract

Manganese (Mn), iron (Fe), copper (Cu), zinc (Zn), and selenium (Se) are essential micronutrients for human health. However, the genetic basis for the content of Mn, Fe, Cu, Zn, and Se in wheat grains remains unclear. A recombinant inbred lines (RIL) population derived from Yangmai 4/Yanzhan 1 (YM4/YZ1) with wheat 55K single nucleotide polymorphism (SNP) arrays and micronutrient content of two environments was used to construct a genetic linkage map and dissect the quantitative trait loci (QTL) for the content of Mn, Fe, Cu, Zn, and Se in wheat. A total of 8 QTL were detected and located on chromosomes 1A, 1B, 2D, 4D, 7A, and 7D, respectively. Among them, *QFe.yaas-2D* and *QSe.yaas-2D* were co-located on chromosome 2D, while *QMn.yaas-4D* and *QZn.yaas-4D* were co-located on chromosome 4D, which were in the dwarfing locus of *Rht-D1* region. The positive alleles of *QCu.yaas-1A*, *QMn.yaas-1B*, and *QZn.yaas-7D* were contributed by YZ1 and explained 7.66–19.92% of the phenotypic variances, while the positive alleles of *QFe.yaas-2D*, *QSe.yaas-2D*, *QMn.yaas-4D*, *QZn.yaas-4D*, and *QCu.yaas-7A* were contributed by YM4 and explained 5.77–20.11% of the phenotypic variances. The positive alleles of *QCu.yaas-1A*, *QMn.yaas-1B*, and *QMn/Zn.yaas-4D* increased TGW by 3.52%, 3.45%, and 7.51% respectively, while the positive alleles of *QFe/Se.yaas-2D* decreased TGW by 6.45%. Six SNP markers flanked the target QTL were converted into Kompetitive allele specific PCR (KASP) markers, and their effects were validated in a panel of one hundred and forty-nine wheat advanced lines. Twenty-five advanced lines harboring at least five positive alleles were identified in the validation populations. A total of 60 and 51 high-confidence annotated genes for *QFe/Se.yaas-2D* and *QMn/Zn.yaas-4D* were identified using the International Wheat Genome Sequencing Consortium Reference Sequence v2.1 (IWGSC RefSeq v2.1), respectively. Some genes in these two regions were involved in stress tolerance, growth development, Zn synthesis in plants. These results provide the basis for fine-mapping the target QTL of micronutrient content and marker-assisted selection in grain quality breeding programs.

## 1 Introduction

Agricultural products provide the primary source of nutrition for human beings. However, the variety and abundance of micronutrients in major food crops such as wheat, rice, and corn are significantly low (Calloway, 1995). Consequently, lack of micronutrients, particularly zinc (Zn) and iron (Fe), are prevalent in human diets followed by copper (Cu), Manganese (Mn), and selenium (Se) (Whanger, 1989; Yan et al., 2002). Micronutrient deficiencies lead to annual economic losses estimated at 30 billion yuan due to malnutrition, with projected cumulative losses potentially exceeding hundreds of billions of yuan for China in the forthcoming decade (World Bank, 1994; WHO, 1999). The increasing severity of diseases caused by micronutrient deficiency, particularly among infants, women, and the elderly has drawn more and more attention in the world. Mn plays a crucial role in enzyme activity in the human body, contributing to the maintenance of normal brain function as well as proper fat and sugar metabolism. Insufficient Mn leads to anemia, asthma, dwarfism, Parkinson’s syndrome, and other related disease. Fe in the human body is categorized into two forms: functional Fe (including myoglobin iron, transferrin iron, and hemoglobin iron) and stored Fe (comprising hemosiderin and ferritin) (Ceylan et al., 2021; Li et al., 2023; Pootheri et al., 2024). The average individual requires 20-25 mg of Fe daily for hematopoiesis primarily obtained from red blood cell aging and destruction. In addition, Fe also participates in hormone synthesis or enhances hormonal effects, maintains normal immune function of the body, enhances neutrophil bactericidal and phagocytic functions, facilitates common element transport, and maintains the proliferation and differentiation of T and B cells and the production of antibodies (Folin et al., 1991). Zn plays an important role in human growth and development, immune regulation, and vitamin utilization. It is involved in coenzyme formation as well as being a constituent of Zn metal enzymes and Zn lipoproteins. Consequently, many coenzymes contain Zn (Wang et al., 2021). Fe deficiency anemia and Zn deficiency are associated with malnutrition, growth retardation, compromised immunity, and reduced IQ (Wang et al., 2021). Cu is indispensable for human health and plays a crucial role in various physiological functions, including regulation and maintenance of hematopoiesis, enzyme composition and activation, preservation of hair pigment structure, cardiovascular and bone health maintenance, as well as facilitation of growth promotion and development (Durnam and Palmiter, 1981; Ohalloran, 1993). Chronic and severe deficiency of marginal Cu can lead to childhood dysplasia and endemic diseases like African genu varus. Due to its impact on vascular components, leukocyte, and platelet function, as well as lipoprotein metabolism, Cu significantly contributes to the development of atherosclerosis, inadequate Cu intake can result in atherosclerosis in humans (Ferns et al., 1997; Ghayour-Mobarhan et al., 2005). Furthermore, Cu serves as an essential component of human neural substances; insufficient intake may cause disorders within the nervous system leading to insomnia, memory decline, cognitive impairment, and slow reaction time among other symptoms (Bavi et al., 2023). Se is an essential component of glutathione peroxidase, demonstrating potent antioxidant activity and playing a pivotal role in scavenging free radicals and combating the aging process. Hence, Se has the potential to hinder the progression of liver cancer, lung cancer, colon cancer, leukemia, and other refractory diseases. Insufficient levels of Se can weaken enzyme activity and lead to inadequate enzyme synthesis, thereby compromising normal physiological functions and potentially contributing to premature aging (Wei et al., 2020). Most micronutrients are quantitative traits and difficult to be detected due to the big genome of wheat. Previous studies have identified numerous genetic loci for micronutrients through the Genome-Wide Association Study (GWAS) (Hao et al., 2024). Baranwal et al. (2022) found that in addition to enhancing disease resistance, the 1B:1R translocation line can also increase the content of Zn, Fe, Cu, and sulfur in wheat grains, while the 2NS translocation line can increase the content of Fe and Cu. Additionally, correlations of micronutrient content and yield-related traits have been seldom reported. The rapid development of molecular marker-assisted technology offers an efficient tool for revealing the genetic mechanisms of quantitative traits. Application of molecular markers linking with major loci/genes in breeding is common. Currently, single nucleotide polymorphisms (SNP) have largely replaced simple sequence repeats (SSR) due to their most abundant feature at the genomic level. Kompetitive allele-specific PCR (KASP) enables high-precision allele typing for specified SNPs and InDels (insertions and deletions). Compared to other verification techniques, KASP offers superior analytical stability and accuracy, lower reaction costs, and higher throughput, making it a cost-effective genotyping technique (Chen and Sullivan, 2003; Yu et al., 2014).

Due to being paid less attention, only a few quantitative trait loci (QTL) for grain micronutrient content with stable main effects were identified and validated in previous studies, while the molecular markers associated with QTL were even rare (Wei et al., 2020). Yangmai 4 (YM4) is a high-yield and disease-resistant wheat variety released in the Middle and Lower reaches of the Yangtze River (MLYR). Yanzhan 1 (YZ1) is a high-yielding and early-maturing wheat variety released in the Huang-Huai Wheat Region (HWR). Previously, the whole-genome genetic map of the YM4/YZ1 population was built by Hu et al (Hu et al., 2023c). In this study, we use 151 YM4/YZ1 F_6_ recombinant inbred lines (RIL) to (1) explore the genetic basis of Mn, Fe, Cu, Zn, and Se content in YM4 and YZ1; (2) quantify the effect of the micronutrient QTL on corresponding traits and thousand grain weight (TGW) in the mapping population; (3) develop breeder-friendly molecular markers (KASP) closely linked to the target region; and (4) assess the effect of the target QTL in wheat advanced lines.

## 2 Materials and methods

### 2.1 Plant materials and field tests

A F_6_ population comprising 151 RIL, derived from a cross between YM4 and YZ1 was developed by a single seed descent approach at the Institute of Crop Sciences, Chinese Academy of Agricultural Sciences (CAAS). These RIL populations were grown at Wanfu Experimental Base of Lixiahe Institute of Agricultural Sciences (Yangzhou, Jiangsu) in 2019-2020 (2020YZ) and 2020-2021 (2021YZ). Field experiments were conducted in a completely randomized design with two replications. For each RIL and cultivar, an average of fifty seeds per row were sown in 180-cm double rows separated 25 cm apart. Fertilization and field management should adhere to local agricultural practices, ensure timely pest and disease control, and manually harvest each variety at maturity according to the designated plot. In 2021, wheat grains were harvested at their respective maturity stages. After harvest, the healthy seeds were measured for thousand grain weight using the Wanshen SC-G seed detector. Afterward, the wheat seeds underwent three rounds of washing with deionized water, followed by drying, baking in a constant temperature box until reaching a constant weight, and grinding through a sample sieve for subsequent analysis and testing. One hundred and forty-nine wheat advanced lines developed by the Lixiahe Institute of Agricultural Sciences were grown in 2022 using the same protocol as RIL (**Supplementary Table 1**).

### 2.2 Phenotype determination

The analysis of micronutrients was conducted by Nanjing Yizhiyuan Testing Technology Co., Ltd. The determination of Mn, Fe, Cu, and Zn involves weighing a 0.2 g sample (with an accuracy of 0.0001 g) and placing it in a PTFE tank. Then, 5 mL of concentrated nitric acid and 2 mL of hydrogen peroxide were added into the tank. The mixture is placed on a graphene electric heating plate and heated at 150°C for digestion. Nitric acid is added midway until the digestion solution becomes transparent. The temperature is then set to 170°C to evaporate excess acid until approximately 1 mL of solution remains. After cooling with pure water up to a volume of 50 mL, the solution is filtered for analysis using a Thermo Fisher double channel inductively coupled plasma emission spectrometer (ICP-AES) to determine the content of Mn, Fe, Cu, and Zn. Two replicates were set up for each experiment and were conducted simultaneously. The mixed standard solution was injected into ICP-AES, and the signal response values of the element and the internal standard element were determined. The standard curve was drawn with the concentration of the element as the abscissa and the ratio of the response signal value of the element to the selected internal standard element as the ordinate. The blank solution and the sample solution were injected into ICP-AES, and the signal response values of the element and the internal standard element were determined. The concentration of the element in the digestion solution was obtained according to the standard curve. The content of the element to be examined in the sample:


$$\text{X}=\frac{\text{P}-{\text{P}}_{0}\times \text{V}\times \text{f}}{\text{m}}$$

Where: X-element content in the sample (mg/kg); P-element mass concentration in the sample solution (mg/L); P_0_-element mass concentration in the blank solution of the sample (mg/L); V-constant volume of sample digestive liquid (mL); f-dilution factor of the sample; m-weighing mass of the sample (g).

Determination of Se: weigh 0.5 g sample (accurate to 0.0001 g), place in a PTFE tank, add 5 mL concentrated nitric acid, 2 mL hydrogen peroxide, place on the graphene electric heating plate, 150°C heating digestion, midway add nitric acid until the solution is transparent, set 170°C to chase acid to the solution remaining about 1 mL, cooling with pure water to 50 mL, filter, atomic fluorescence spectrometer (AFS-933) on the machine determination of Se content. The blank test was also conducted. The mixed standard solution was injected into the atomic fluorescence spectrometer to determine the signal response values of Se and internal standard elements. The concentration of Se was plotted on the abscissa, while the ratio of the response signal value of Se to the selected internal standard element served as the ordinate for drawing a standard curve. Subsequently, both the blank solution and sample solution were injected into the atomic fluorescence spectrometer to determine their respective signal response values for Se and internal standard elements. Finally, based on the obtained standard curve, the concentration of Se in the digestion solution was determined. The content of Se in the sample is calculated by the formula above.

### 2.3 Data statistics

The initial data were processed and statistically analyzed using SPSS 22.0 and Microsoft Excel 2019, primarily including descriptive statistics and t-tests. The analysis of variance (ANOVA) was estimated using IciMapping v4.1, and the broad-sense heritability ( ${h_{b}}^{2}$ ) for micronutrients was estimated using the formula ${h_{b}}^{2}={\sigma^{2}}_{G}/({\sigma^{2}}_{G}+{\sigma^{2}}_{G\times E}/E+{\sigma^{2}}_{e}/ER)$ , where E and R represent the number of environments and replicates, respectively, ${\sigma^{2}}_{G}$ is the genotypic effect, ${\sigma^{2}}_{G\times E}$ is the effect of genotype by the environment, and ${\sigma^{2}}_{e}$ is the residual error (Nyquist and Baker, 1991).

### 2.4 QTL mapping

The genomic DNA of the tested materials were extracted by the CTAB method (Stacey and Isaac, 1994). The tested parents and RIL populations were detected for 55 K SNP markers by using the Bead Array technology of the Illumina SNP Genotyping technology test platform (Beijing BOA Biotechnology Co., Ltd.), and the corresponding KASP markers including *Rht-B1*, *Rht-D1* and *TaGW2-6A* genes that control important traits of wheat were integrated into the polymorphic markers (Rasheed et al., 2016). After filtering and screening all genotypes of polymorphic markers, a total of 7974 polymorphic SNP markers were obtained. After removing the redundancy, 1546 SNPs were obtained. Finally, 1440 SNPs were used to construct a genetic linkage map covering 21 chromosomes of wheat with a length of 3574.10 cM. The inclusive composite interval mapping (ICIM) was employed to identify QTL significantly associated with grain micronutrient content in wheat, with a LOD threshold set at 2.5 (Li et al., 2021). Genetic maps for each chromosome were drawn using MapChart 2.3 software (Hu et al., 2023b). QTL exhibiting a genetic position peak within 10 cM on the same chromosome were considered identical, and the nomenclature of QTL was followed by Hu et al. (2023a). To determine the novel loci identified in this study, we compared the flanking markers sequences of these loci with those of previously reported loci in the EnsemblPlants database (http://plants.ensembl.org/).

### 2.5 Marker development and QTL validation in different genetic backgrounds

KASP markers were developed using the flanking SNP of the target QTL according to the method of Xu et al. (2019) (PolyMarker, http://polymarker.tgac.ac.uk/). Specific sequences that can bind to FAM fluorescence were added to the tail of the F1 primer, and specific sequences that can bind to HEX fluorescence were added to the tail of the F2 primer, synthesized by Beijing Jiacheng Biotechnology Co., Ltd. A total of one hundred and forty-nine wheat advanced lines were used for KASP marker validation. The PCR reaction was performed on an ABI Veriti 384 PCR instrument (Thermo Fisher). The PCR amplification products were scanned, and fluorescence values were obtained using the Omega F SNP typing detector (LGC Genomics Ltd, KBS-0024-002). Kluster CallerTM (KBioscience) software was used for genotyping analysis.

### 2.6 Prediction of candidate genes for major QTL

Genes and their annotations within the mapping intervals were extracted according to International Wheat Genome Sequencing Consortium Reference Sequence v2.1 (IWGSC RefSeq v2.1) (http://202.194.139.32/jbrowse-1.12.3-release/?data=Chinese_Spring2.1&loc) (Hu et al., 2022). To further define the biological process and molecular function of the candidate genes, the description and GO enrichment were also analyzed using Triticeae-GeneTribe (http://wheat.cau.edu.cn/TGT/) (Zhao et al., 2023).

## 3 Results

### 3.1 Phenotypic analysis

The Fe, Cu, Zn, and Se content of YM4 were significantly higher than those of YZ1, and the Mn content of YZ1 was significantly higher than that of YM4 (**Table 1**). In the RIL population, Mn content ranged from 2.11 to 25.63 mg/kg, Fe content from 10.97 to 76.34 mg/kg, Cu content from 0.01 to 4.82 mg/kg, Zn content from 16.02 to 67.82 mg/kg, and Se content from 0.01 to 0.15 mg/kg based on the mean datasets of two trials (**Table 1**). A pattern of continuous distribution for each micronutrient was observed in each environment and the mean datasets in the RIL population. Heritability of Mn, Fe, Cu, Zn, and Se content in RIL population were 0.85, 0.87, 0.93, 0.69, and 0.89, respectively (**Table 1**).

**Table 1:** Phenotypic variation of content of micronutrients in the parents and RIL population of Yangmai 4/Yanzhan 1.

Trait	Environment	YM4	YZ1	Population					Heritability
				Maximum	Minimum	Mean	Skewness	Kurtosis	
Mn content (mg/kg)	2020	20.81 ± 1.09	26.78 ± 1.39*	22.44	2.32	9.45	1.05	1.14	0.85
	2021	22.87 ± 1.23	27.15 ± 1.67*	29.87	0.32	7.53	1.63	2.73	
	Mean	21.84	26.97	26.16	2.97	8.49	1.78	3.18	
Fe content (mg/kg)	2020	31.03 ± 1.39	21.06 ± 1.04*	70.89	9.50	26.20	1.50	3.36	0.87
	2021	32.46 ± 1.44	23.87 ± 1.23*	88.12	10.11	32.91	0.96	0.47	
	Mean	31.75	22.46	78.91	11.50	29.55	1.09	0.91	
Cu content (mg/kg)	2020	3.48 ± 0.65	2.53 ± 0.49**	3.96	0.01	1.55	0.65	0.50	0.93
	2021	4.52 ± 0.78	2.66 ± 0.38**	5.99	0.01	2.37	0.59	0.11	
	Mean	4.00	2.60	9.64	0.02	3.92	0.63	0.26	
Zn content (mg/kg)	2020	22.61 ± 0.98	15.66 ± 0.83*	45.14	20.20	26.81	1.49	2.36	0.69
	2021	23.76 ± 1.42	16.14 ± 0.59*	90.50	10.23	28.81	1.33	1.83	
	Mean	23.19	15.90	135.64	32.03	55.62	1.43	2.55	
Se content (mg/kg)	2020	0.08 ± 0.01	0.02 ± 0.01*	0.12	0.01	0.05	0.38	-0.44	0.89
	2021	0.14 ± 0.01	0.04 ± 0.01*	0.19	0.01	0.06	0.96	0.97	
	Mean	0.11	0.03	0.14	0.01	0.06	0.60	0.17	
TGW (g)	2021	49.55 ± 2.36	43.48 ± 1.90*	58.09	32.60	43.74	0.55	0.31	

* indicate the significant levels at *P* < 0.05, ** indicate the significant levels at *P* < 0.01. TGW, thousand grain weight.

In the YM4/YZ1 RIL population, Pearson correlation analysis was conducted for all traits based on the mean datasets across different environments. The results indicated significant positive correlations were observed between Mn and Fe (*r* = 0.370, *P* < 0.01), Mn and Cu (*r* = 0.501, *P* < 0.01), Mn and Zn (*r* = 0.481, *P* < 0.01), and Mn and TGW (*r* = 0.280, *P* < 0.01) (**Table 2**). Additionally, Fe showed significant positive correlations with Cu (*r* = 0.426, *P* < 0.01), Zn (*r* = 0.415, *P* < 0.01), and TGW (*r* = 0.235, *P* < 0.01) (**Table 2**). Cu was found to be significantly and positively correlated with Zn (*r* = 0.409, *P* < 0.01) and TGW (*r* = 0.206, *P* < 0.01) (**Table 2**). Furthermore, Zn exhibited significant positive correlations with Se (*r* = 0.170, *P* < 0.05) and TGW (*r* = 0.310, *P* < 0.01) (**Table 2**).

**Table 2:** Pearson correlation coefficients among TGW and content of Mn, Fe, Cu, Zn, Se at the Yangmai 4/Yanzhan 1 RIL population.

Trait	Mean-Mn	Mean-Fe	Mean-Cu	Mean-Zn	Mean-Se	Mean-TGW
Mean-Mn	–					
Mean-Fe	0.370**	–				
Mean-Cu	0.501**	0.426**	–			
Mean-Zn	0.481**	0.415**	0.409**	–		
Mean-Se	-0.018	0.131	0.060	0.170*	–	
Mean-TGW	0.280**	0.235**	0.206*	0.310**	-0.009	–

* and **, significant at the 0.05 and 0.01 probability level, respectively; TGW, thousand grain weight.

### 3.2 QTL mapping for grain micronutrients

**Figure 1:**
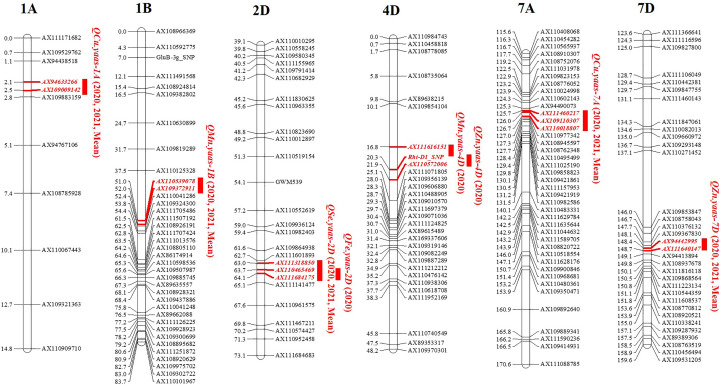
QTL mapping for content of micronutrients in the RIL population. Markers’ names are shown on the right of linkage groups, and their genetic positions are shown on the left (cM). The names of QTL are shown on the right side of the markers. 2020, 2021 and Mean represent environments of 2020, 2021 and mean value, respectively.

Among all the detected QTL for micronutrients, *QMn.yaas-1B*, *QCu.yaas-1A*, *QCu.yaas-7A*, *QZn.yaas-7D*, and *QSe.yaas-2D* could be detected in two environments and mean values, while *QMn.yaas-4D*, *QFe.yaas-2D*, and *QZn.yaas-4D* could be detected in only one environment (**Table 3**). Two QTL for Mn were identified on chromosomes 1B and 4D, and the positive allele of *QMn.yaas-1B* was derived from YZ1, explaining 10.16% to 18.04% of phenotypic variances, while the positive allele of *QMn.yaas-4D* was derived from YM4, explaining 6.02% of phenotypic variances (**Table 3**). One QTL *QFe.yaas-2D* for Fe was identified on chromosome 2D, and the positive allele of it is from YM4, explaining 8.04% of the phenotypic variances (**Table 3**). Two QTL for Cu were identified on chromosomes 1A and 7A, and the positive allele of *QCu.yaas-1A* is from YZ1, explaining 15.87%–19.92% of the phenotypic variances, while the positive allele of *QCu.yaas-7A* is from YM4, explaining 5.77%–10.59% of the phenotypic variance (**Table 3**). Two QTL for Zn were detected on chromosomes 4D and 7D, and the positive allele of *QZn.yaas-4D* is from YM4 with 12.29% of phenotypic variances, while the positive allele of *QZn.yaas-7D* is from YZ1, explaining 7.66%–11.26% of phenotypic variances (**Table 3**). One QTL *QSe.yaas-2D* for Se was identified on chromosome 2D, and the positive allele of it is from YM4, explaining 11.52% to 20.11% of phenotypic variances (**Table 3**, **Figure 1**).

**Table 3:** QTL for content of micronutrients in different environments.

Trait	QTL	Environment	Genetic position (cM)	Physical position (Mb)	Marker interval	LOD	PVE^a^ (%)	Add^b^
Mn	*QMn.yaas-1B*	2020	51.00	45.15–52.85	AX110539078–AX109372911	8.23	18.04	-1.63
		2021	51.50	45.15–52.85	AX110539078–AX109372911	4.43	10.16	-1.81
		Mean	51.09	45.15–52.85	AX110539078–AX109372911	8.15	17.49	-1.80
Fe	*QMn.yaas-4D*	2020	16.80	16.58–19.19	AX111616151–Rht-D1_SNP	2.98	6.02	1.13
	*QFe.yaas-2D*	2020	64.00	541.93–543.82	AX110465469–AX111684175	2.75	8.04	3.14
Cu	*QCu.yaas-1A*	2020	2.40	466.52–481.15	AX94633266–AX109009142	7.98	15.87	-0.29
		2021	2.40	466.52–481.15	AX94633266–AX109009142	6.42	16.55	-0.50
		Mean	2.40	466.52–481.15	AX94633266–AX109009142	8.52	19.92	-0.63
	*QCu.yaas-7A*	2020	125.70	621.57–632.35	AX111460217–AX109110307	3.12	5.77	0.18
		2021	126.06	632.35–641.13	AX109110307–AX110018807	3.47	8.55	0.36
		Mean	125.90	621.57–632.35	AX111460217–AX109110307	3.62	10.59	0.53
Zn	*QZn.yaas-4D*	2020	20.70	19.19–19.59	Rht-D1_SNP–AX110572006	5.46	12.29	2.06
	*QZn.yaas-7D*	2020	148.70	551.35–551.52	AX94442995–AX111640147	3.59	7.66	-1.35
		2021	148.56	551.35–551.52	AX94442995–AX111640147	3.64	10.08	-4.87
		Mean	148.70	551.35–551.52	AX94442995–AX111640147	3.98	11.26	-4.99
Se	*QSe.yaas-2D*	2020	63.50	539.51–541.93	AX111318850–AX110465469	6.98	20.11	11.67
		2021	64.10	541.93–543.82	AX110465469–AX111684175	4.01	11.52	11.84
		Mean	64.00	541.93–543.82	AX110465469–AX111684175	6.37	18.18	11.37

^a^ PVE, phenotypic variance explained. ^b^ Positive additive effect indicates that the increasing effect were contributed by Yangmai 4, the negative additive effect indicates that the increasing effect were contributed by Yanzhan 1.

### 3.3 Micronutrients QTL effect on thousand grain weight

**Figure 2:**
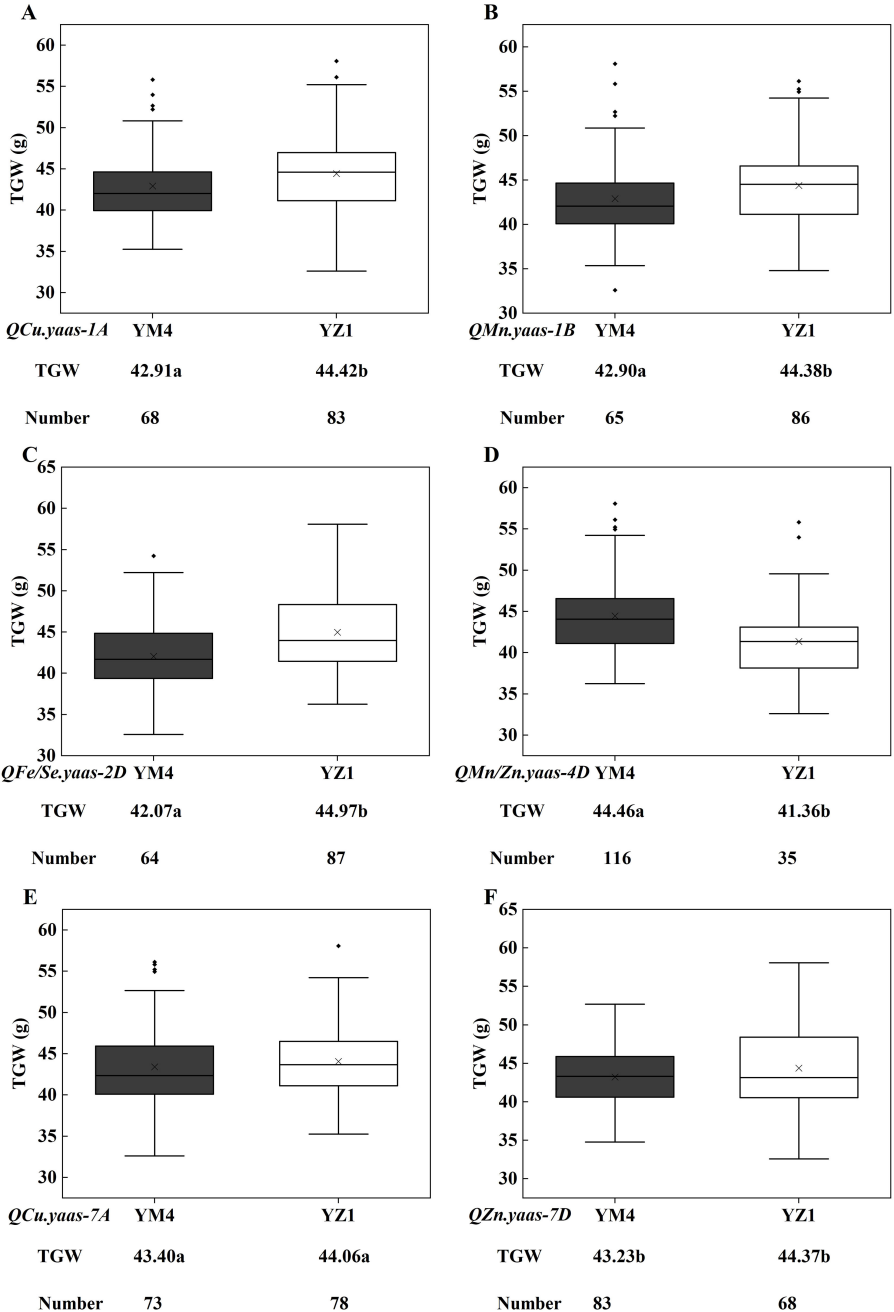
Allelic effects of different micronutrient QTL on TGW in the Yangmai 4/Yanzhan 1 RIL population. **(A)** *QCu.yaas-1A*. **(B)** *QMn.yaas-1B*. **(C)** *QFe/Se.yaas-2D*. **(D)** *QMn/Zn.yaas-4D*. **(E)** *QCu.yaas-7A*. **(F)** *QZn.yaas-7D*. TGW, thousand grain weight. ‘×’ in the data box indicates the mean value; the dots in the boxplots are the outliers; the horizontal line in the data box indicates the median. Values followed by different letters represent significant difference in TGW between genotypes (*P*<0.05). Values followed by the same letter represent no significant difference in TGW between genotypes.

The flanking linkage markers AX109009142 (1A), AX109372911 (1B), AX111684175 (2D), AX110572006 (4D), AX109110307 (7A), and AX111640147 (7D) for *QCu.yaas-1A*, *QMn.yaas-1B*, *QFe/Se.yaas-2D*, *QMn/Zn.yaas-4D*, *QCu.yaas-7A*, and *QZn.yaas-7D* were used to evaluate their effects on TGW in the YM4/YZ1 population. For *QCu.yaas-1A* and *QMn.yaas-1B*, the YZ1 homozygous allele increased TGW by 3.52% (*P*<0.05) and 3.45% (*P*<0.05) relative to the YM4 homozygous allele, respectively (**Figures 2A, B**). For *QFe/Se.yaas-2D*, the YM4 homozygous allele decreased TGW by 6.45% (*P*<0.001) relative to the YZ1 homozygous allele (**Figure 2C**). For *QMn/Zn.yaas-4D*, the YM4 homozygous allele increased TGW by 7.51% (*P*<0.001) relative to the YZ1 homozygous allele (**Figure 2D**). *QCu.yaas-7A* and *QZn.yaas-7D* did not significantly affect TGW (**Figures 2E, F**).

### 3.4 Development and validation of KASP markers for target QTL

**Figure 3:**
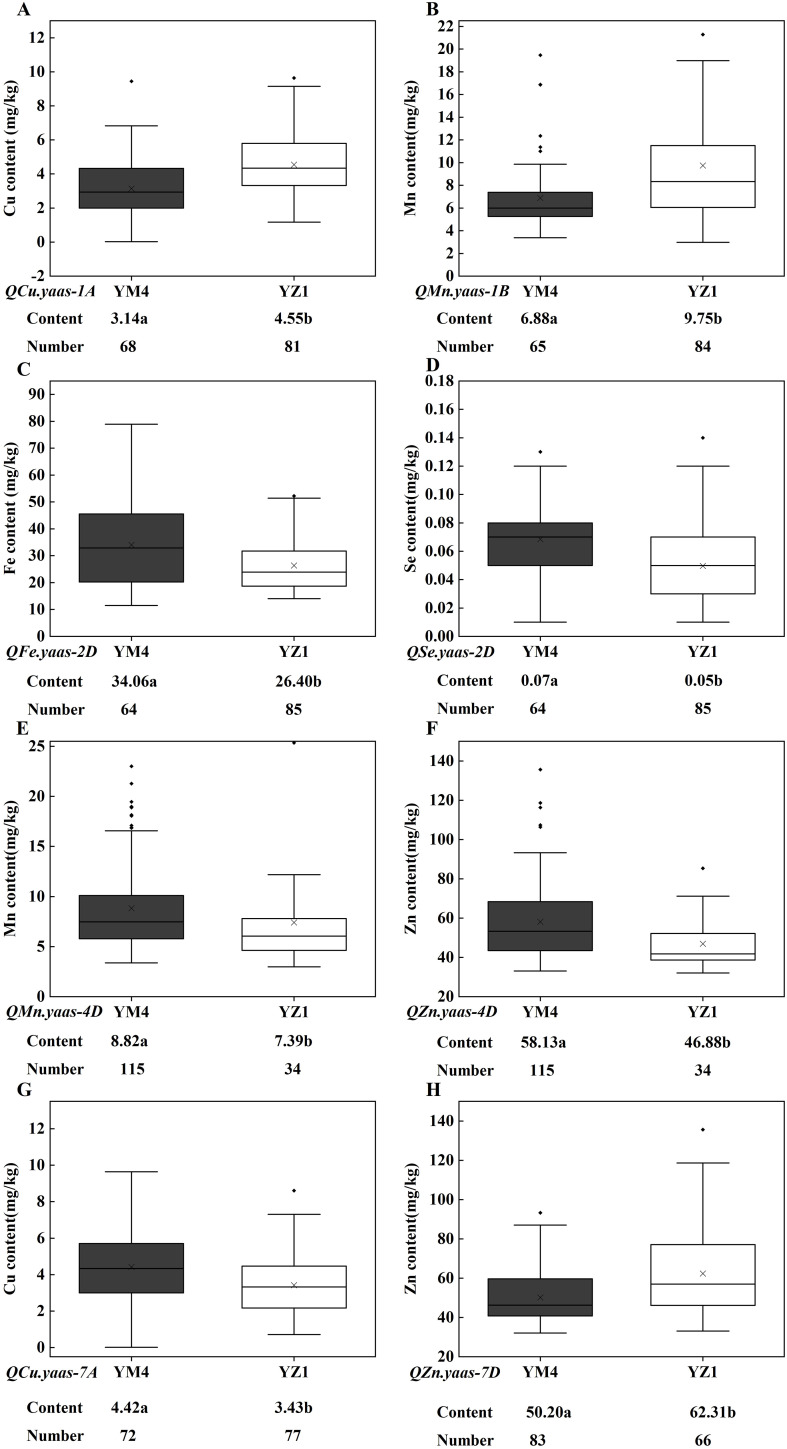
Allelic effects of KASP markers on the content of Cu, Mn, Fe, Zn and Se in the advanced wheat line. **(A)** *QCu.yaas-1A*. **(B)** *QMn.yaas-1B*. **(C)** *QFe.yaas-2D*. **(D)** *QSe.yaas-2D*. **(E)** *QMn.yaas-4D*. **(F)** *QZn.yaas-4D*. **(G)** *QCu.yaas-7A*. **(H)** *QZn.yaas-7D*. ‘×’ in the data box indicates the mean value; the dots in the boxplots are the outliers; the horizontal line in the data box indicates the median. Values followed by different letters represent significant difference in micronutrient content between genotypes (*P*<0.05).

The SNP markers AX109009142 (1A), AX109372911 (1B), AX111684175 (2D), AX110572006 (4D), AX109110307 (7A), and AX111640147 (7D), which linked with the target QTL in the current study were finally converted into KASP markers and designated as KASP.1A, KASP.1B, KASP.2D, KASP.4D, KASP.7A and KASP.7D (**Table 4**). The KASP markers were used to screen one hundred and forty-nine wheat advanced lines planted in 2022 in the Yangzhou yield evaluation nursery to verify the target QTL effect on wheat grain micronutrient content. For *QCu.yaas-1A*, the lines carrying the YZ1 allele increased Cu content by 44.90% relative to those carrying the YM4 allele (*P*<0.001) (**Table 5**, **Figure 3A**). For *QMn.yaas-1B*, the lines carrying the YZ1 allele increased Mn content by 41.72% relative to those carrying the YM4 allele (*P*<0.001) (**Table 5**, **Figure 3B**). For *QFe.yaas-2D*, the lines carrying the YM4 allele increased Fe content by 29.02% relative to those carrying the YZ1 allele (*P*<0.001) (**Table 5**, **Figure 3C**). For *QSe.yaas-2D*, the lines carrying the YM4 allele increased Se by 40% relative to the lines carrying the YZ1 allele (*P*<0.001) (**Table 5**, **Figure 3D**). For *QMn.yaas-4D*, the lines carrying the YM4 allele increased Mn content by 19.35%relative to those carrying the YZ1 allele (*P*<0.05) (**Table 5**, **Figure 3E**). For *QZn.yaas-4D*, the lines carrying the YM4 allele increased Zn content by 24.00% relative to those carrying the YZ1 allele (*P*<0.001) (**Table 5**, **Figure 3F**). For *QCu.yaas-7A*, the lines carrying the YM4 allele increased Cu content by 28.86% relative to those carrying the YZ1 allele (*P*<0.001) (**Table 5**, **Figure 3G**). For *QZn.yaas-7D*, the lines carrying the YZ1 allele increased Zn content by 24.12% relative to those carrying the YM4 allele (*P*<0.001) (**Table 5**, **Figure 3H**).

**Table 4:** Protocols for assaying markers.

Marker	Primer (5’–3’) ^a^
KASP.1A	F1: GAAGGTGACCAAGTTCATGCTagtgagtactgccactccaA
	F2: GAAGGTCGGAGTCAACGGATTagtgagtactgccactccaG
	R: cctagtagtagagcaggtggtG
KASP.1B	F1: GAAGGTGACCAAGTTCATGCTgcattgaggtttcacgtagactT
	F2: GAAGGTCGGAGTCAACGGATTgcattgaggtttcacgtagactC
	R: cgagcgtctacatctactttgt
KASP.2D	F1: GAAGGTGACCAAGTTCATGCTaaggggaaacatcatattcttccT
	F2: GAAGGTCGGAGTCAACGGATTaaggggaaacatcatattcttccC
	R: gtagcacgtcactgtcgaga
KASP.4D	F1: GAAGGTGACCAAGTTCATGCTtgttgaatcggtaaaagggcG
	F2: GAAGGTCGGAGTCAACGGATTtgttgaatcggtaaaagggcA
	R: gggtttagctacaaggcatgg
KASP.7A	F1: GAAGGTGACCAAGTTCATGCTgtttgccatcatatcaaacctcG
	F2: GAAGGTCGGAGTCAACGGATTgtttgccatcatatcaaacctcT
	R: cagaggcacgtcatcgacat
KASP.7D	F1: GAAGGTGACCAAGTTCATGCTgcaacaccaggaatttcatccaC
	F2: GAAGGTCGGAGTCAACGGATTgcaacaccaggaatttcatccaT
	R: tcccggttctgttagggtca

a F1 and F2 are two forward primers, R is the reverse primer. The tails for two competitive primers are underlined.

**Table 5:** Allelic effects of micronutrient QTL on corresponding trait in the one hundred and forty-nine wheat lines.

	Allele	Mn (mg/kg)	Fe (mg/kg)	Cu (mg/kg)	Zn (mg/kg)	Se (mg/kg)	Number
*QCu.yaas-1A*	YM4 allele	–	–	3.14	–	–	68
	YZ1 allele	–	–	4.55***	–	–	81
*QMn.yaas-1B*	YM4 allele	6.88	–	–	–	–	65
	YZ1 allele	9.75***	–	–	–	–	84
*QFe.yaas-2D*	YM4 allele	–	34.06	–	–	–	64
	YZ1 allele	–	26.40***	–	–	–	85
*QSe.yaas-2D*	YM4 allele	–	–	–	–	0.07	64
	YZ1 allele	–	–	–	–	0.05***	85
*QMn.yaas-4D*	YM4 allele	8.82	–	–	–	–	115
	YZ1 allele	7.39*	–	–	–	–	34
*QZn.yaas-4D*	YM4 allele	–	–	–	58.13	–	115
	YZ1 allele	–	–	–	46.88***	–	34
*QCu.yaas-7A*	YM4 allele	–	–	4.42	–	–	72
	YZ1 allele	–	–	3.43***	–	–	77
*QZn.yaas-7D*	YM4 allele	–	–	–	50.20	–	83
	YZ1 allele	–	–	–	62.31***	–	66

* indicate the significant levels at *P* < 0.05, *** indicate the significant levels at *P* < 0.001, compared with YM4 allele. YM4 indicates Yangmai 4 allele, and YZ1 indicates Yanzhan 1 allele.

### 3.5 Identification of lines with positive alleles at micronutrients

Among the one hundred and forty-nine wheat advanced lines, eight lines pyramiding six positive alleles at all loci, with the Mn content ranging from 5.72 mg/kg to 21.28 mg/kg; the Fe content ranging from 24.87 mg/kg to 69.28 mg/kg; the Cu content ranging from 3.46 mg/kg to 9.15 mg/kg; the Zn content ranging from 37.52 mg/kg to 116.29 mg/kg; the Se content ranging from 0.02 mg/kg to 0.12 mg/kg (**Table 6**). The average micronutrient values of these eight lines were 13.24 mg/kg for Mn, 41.63 mg/kg for Fe, 6.49 mg/kg for Cu, 68.57 mg/kg for Zn, and 0.07 mg/kg for Se. Seventeen lines harbored five positive alleles, with average Mn of 10.99 mg/kg, Fe of 39.70 mg/kg, Cu of 5.30 mg/kg, Zn of 68.59 mg/kg, and Se of 0.06 mg/kg. These twenty-five lines, along with the KASP markers linked with the micronutrient content QTL, will be used in wheat breeding for quality.

**Table 6:** Genotypes and phenotypes of 25 advanced lines carrying more than 5 QTL synergistic alleles.

Advanced line	Genotype						Phenotype mg/kg				
	KASP.1A	KASP.1B	KASP.2D	KASP.4D	KASP.7A	KASP.7D	Mean-Mn	Mean-Fe	Mean-Cu	Mean-Zn	Mean-Se
Line 9	B	B	A	A	A	B	18.89	42.16	7.47	79.55	0.07
Line 19	B	B	A	A	A	B	21.28	60.08	8.47	116.29	0.12
Line 21	B	B	A	A	A	B	9.11	33.89	3.46	40.14	0.03
Line 24	B	B	A	A	A	B	9.61	32.56	7.85	55.38	0.07
Line 37	B	B	A	A	A	B	18.06	69.28	9.15	106.36	0.06
Line 57	B	B	A	A	A	B	7.04	36.85	7.04	53.77	0.09
Line 86	B	B	A	A	A	B	5.72	33.37	4.14	37.52	0.09
Line 104	B	B	A	A	A	B	16.22	24.87	4.34	59.53	0.02
Line 7	B	B	A	A	B	B	16.19	35.55	5.89	77.76	0.03
Line 13	B	B	A	A	B	B	13.77	31.13	3.50	59.55	0.08
Line 14	B	B	B	A	A	B	14.10	31.00	7.20	81.04	0.04
Line 15	B	B	B	A	A	B	12.17	38.58	9.64	118.66	0.07
Line 25	B	B	A	A	A	A	13.87	68.51	5.91	66.33	0.10
Line 30	B	B	A	A	B	B	7.65	37.64	3.32	42.20	0.08
Line 33	B	B	A	A	B	B	23.01	66.24	8.61	135.64	0.08
Line 34	B	B	A	A	A	A	8.22	46.70	5.07	51.10	0.03
Line 35	B	B	B	A	A	B	4.75	52.26	4.40	58.89	0.05
Line 43	B	B	A	A	A	A	8.43	47.74	5.57	93.31	0.07
Line 47	B	B	B	A	A	B	8.21	51.38	4.68	66.15	0.03
Line 49	B	A	A	A	A	B	9.85	53.83	6.04	44.32	0.02
Line 76	B	B	A	B	A	B	7.80	16.96	5.89	41.53	0.09
Line 93	B	B	B	A	A	B	9.19	17.91	2.64	63.19	0.06
Line 102	B	B	B	A	A	B	6.26	31.89	3.45	47.49	0.07
Line 129	B	B	B	A	A	B	5.22	25.14	2.70	41.97	0.04
Line 146	B	B	A	A	B	B	18.14	22.48	5.55	76.92	0.12

A indicates Yangmai 4 allele; B indicates Yanzhan 1 allele.

### 3.6 Prediction of candidate genes for *QFe/Se.Yaas-2D* and *QMn/Zn.Yaas-4D*

A total of 60 annotated high-confidence genes (*TraesCS2D03G0955600* to *TraesCS2D03G0967800*) were identified in the target interval of the *QFe/Se.yaas-2D* (**Supplementary Table 2**). These genes predominantly include were mainly acyclic sesquiterpene synthase, probable cation transporter HKT7, Protein FORGETTER 1, and UPF0481 protein At3g47200, etc. (**Supplementary Table 2**). A total of 51 annotated high-confidence genes (*TraesCS4D03G0057000* to *TraesCS4D03G0065300*) were identified in the genomic region of *QMn/Zn.yaas-4D*, mainly encoding Adenylosuccinate synthetase 2, Senescence-specific cysteine protease SAG39, Zinc finger CCCH domain-containing protein 24 and Zinc finger protein 593 homolog, etc. (**Supplementary Table 3**). GO enrichment analysis indicated that these candidate genes of *QFe/Se.yaas-2D* are primarily involved in positive regulation of cellular response to heat, histone binding, and regulation of gene expression, epigenetic (**Supplementary Table 4**, **Supplementary Figure 1**). Several candidate genes of *QMn/Zn.yaas-4D* involved in biological processes of cell development, ethylene responsive, and senescence-associated vacuole (**Supplementary Table 4**, **Supplementary Figure 2**).

## 4 Discussion

### 4.1 Comparison of QTL with previous studies

Rare studies focused on the genetic base of micronutrients in wheat grains. Graham, (1987, 2001) and Schlegel et al. (1991) identified a Cu content QTL on chromosome 5B. Pei (2016) identified the associated loci related to Se content in wheat on chromosomes 3D and 5A. QTL related to Fe content were located on chromosomes 2A and 7A, while QTL related to Se content in wheat grains was located on chromosome 7A (Tiwari et al., 2009). Tong et al. (2022) detected SNP significantly associated with Zn content in wheat grains on chromosomes 1A, 2A, 3A, 3B, 5A, 5D, 6A, 6B, 6D, 7A, 7B, and 7D through GWAS, and also identified SNP significantly associated with Fe content on chromosomes 1A, 1B, 5A, 5B, 7A, 7B and 7D. The above loci were not in the same physical intervals as the detected QTL in this study. Previous studies have shown that there may be a close relationship between different elements, and grain micronutrients have pleiotropic effects on other agronomic traits (Pei, 2016). The *QFe.yaas-2D* and *QZn.yaas-2D* identified in this study were found to be located within the same physical interval that exhibited a significant correlation with Fusarium head blight (FHB) resistance in our previous study (Hu et al., 2023b). Previous studies also indicated a relationship between Se content in wheat grains and FHB resistance (Mao et al., 2020a, b). *QMn. yaas-4D* and *QZn. yaas-4D* co-localize within the same physical region as well as in the wheat dwarf gene *Rht-D1*. Previous studies have demonstrated that *Rht-D1* locus regulated Zn and Mn accumulation in maize grains (Zhou et al., 2010). It is probable that the accumulation of Mn and Zn in grains has a similar genetic basis, while the accumulation of these two micronutrients in wheat grains may be related to the signal transduction of gibberellin.

### 4.2 Utilization of micronutrient QTL in wheat breeding

Hu et al. (2023a) located *QTgw.yas-2DL* (529.23-537 Mb) and *QTgw.yas-4DS* (16.58-19.19 Mb) on chromosomes 2D and 4D, respectively, which are within the same confidence interval as the *QFe/Se.yaas-2D* and *QMn/Zn.yaas-4D* identified in this study. The interaction between the wheat gene network and external environmental factors results in a positive correlation between micronutrients such as manganese and iron and the thousand grain weight phenotype. The pleiotropic effects of QTL on thousand grain weight indicate that the increasing allele of *QFe/Se.yaas-2D* has a negative impact on TGW, while the increasing allele of *QMn/Zn.yaas-4D* has a positive effect on TGW. This suggested that iron or selenium may have an opposite effect on TGW compared to manganese and zinc. Therefore, further fine-mapping of *QFe/Se.yaas-2D*, and *QMn/Zn.yaas-4D* would promote us to analyze the interaction mechanism between micronutrient content and TGW. KASP markers for *QCu.yaas-1A*, *QMn.yaas-1B*, *QMn/Zn.yaas-4D* could be used in wheat breeding for improving micronutrients and TGW.

For one hundred and forty-nine wheat advanced lines, twenty-five lines were found to carry five or more positive alleles at the detected QTL in the current study. The average values for these 25 lines were 11.71 mg/kg for Mn, 40.32 mg/kg for Fe, 5.68 mg/kg for Cu, 68.58 mg/kg for Zn, and 0.06 mg/kg for Se, which increased Mn, Fe, Cu, Zn, and Se by 37.83% (*P*<0.001), 35.80% (*P*<0.001), 45.38% (*P*<0.001), 23.42% (*P*<0.001), and 3.95% (*P*<0.001) relative to the average values of micronutrient content the for the one hundred and forty-nine wheat advanced lines. Therefore, these 25 lines can be used as excellent resources for wheat quality breeding.

### 4.3 Potential candidate for *QFe/Se.yaas-2D* and *QMn/Zn.yaas-4D*

The high-confidence genes within the physical interval of *QFe/Se.yaas-2D* were primarily involved in the synthesis of acyclic sesquiterpene synthase, probable cation transporter HKT7, and UPF0481 protein At3g47200. Acyclic sesquiterpene synthase was associated with the synthesis of gibberellins, which were essential for promoting plant nutrition and reproductive growth, as well as regulating physiological processes and stress responses (Toyomasu et al., 2000). The probable cation transporter HKT7 played a crucial role in regulating ion balance in plants, ensuring their normal growth and development under salt stress conditions (Huang et al., 2006). UPF0481 protein At3g47200 was linked to plant stress tolerance and growth development (https://www.uniprot.org/uniprotkb/A0A1D1Y378/entry). The high-confidence genes in the physical interval of *QMn/Zn.yaas-4D* were mainly related to Adenylosuccinate synthetase 2, Senescence-specific cysteine protease SAG39, and Zinc finger protein 593 homolog. Adenylosuccinate synthetase 2 synthesizes adenylosuccinate in plant chloroplasts, an important intermediate in the purine nucleotide biosynthesis pathway. This enzyme played a crucial role in regulating nucleotide synthesis, cell proliferation, and responses to environmental stress (Zhu et al., 2023). Senescence-specific cysteine protease SAG39 is a protease specifically expressed during plant senescence and played a key role in plant aging and stress responses (Wang et al., 2022). Zinc-finger proteins were characterized by a structural domain that coordinates zinc ions and binds nucleic acids, participating in the regulation of growth, development, and stress adaptation in plants (Han et al., 2021). The results indicated that the aforementioned genes in the *QFe/Se.yaas-2D* and *QMn/Zn.yaas-4D* intervals may be related to stress tolerance, growth development, and zinc synthesis in plants.

## 5 Conclusion

A total of eight QTL associated with micronutrient content were identified in this study. Among them, two QTL related to Mn content, one related to Fe content, two related to Cu content, two related to Zn content, and one related to Sn content were identified. *QFe.yaas-2D* and *QSe.yaas-2D* were co-located. *QMn.yaas-4D* and *QZn.yaas-4D* were co-located at the physical region of *Rht-D1*. Positive alleles of *QCu.yaas-1A*, *QMn.yaas-1B*, and *QMn/Zn.yaas-4D* showed significant favorable effects on TGW. *QFe/Se.yaas-2D* significantly negatively affect TGW. KASP markers *QCu.yaas-1A*, *QMn.yaas-1B*, *QZn.yaas-7D*, *QFe/Se.yaas-2D*, *QMn/Zn.yaas-4D*, and *QCu.yaas-7A* were developed and validated in one hundred and forty-nine wheat advanced lines. Twenty-five lines harboring five or more positive alleles were identified and could be used as excellent resources. A total of 60 and 51 genes were annotated for *QFe/Se.yaas-2D* and *QMn/Zn.yaas-4D*, respectively. This study provides parents and methods for breeding micronutrient-enriched wheat varieties. The detected genetic intervals and candidate genes for *QFe/Se.yaas-2D*, and *QMn/Zn.yaas-4D* will promote further fine-mapping of these two QTL.

## Data Availability

The original contributions presented in the study are included in the article/**Supplementary Material**. Further inquiries can be directed to the corresponding authors.
