# Supplementary material for: Molecular mapping and validation of quantitative trait loci for content of micronutrients in wheat grain
Source: Front Plant Sci. 2025 Jan 17;15:1522465. doi: 10.3389/fpls.2024.1522465 (PMC11782267; doi:10.3389/fpls.2024.1522465)
Supplement: Supplementary file 1 [file DataSheet1.docx]

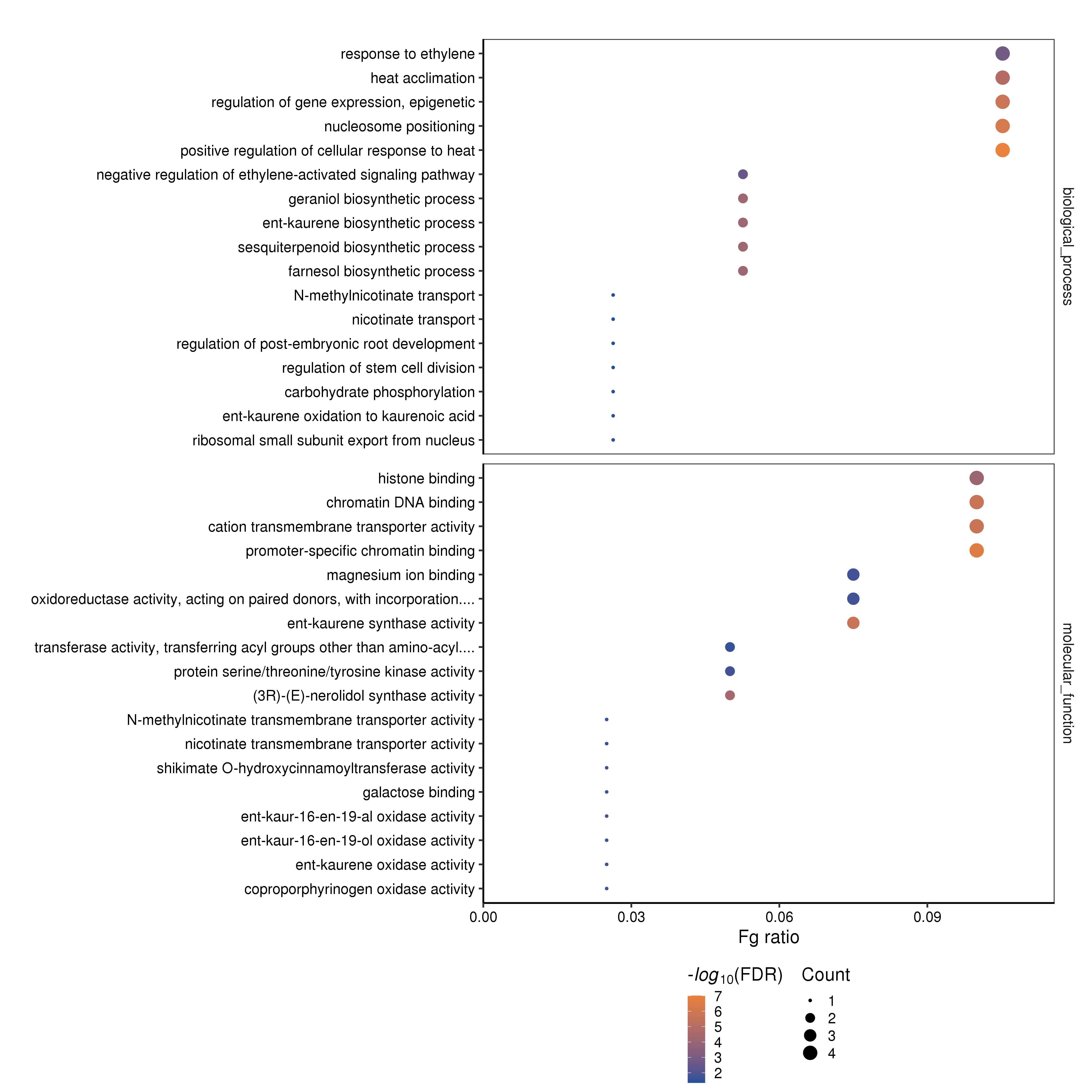


**Supplementary Figure 1** Bubble plots of biological processes and molecular function of GO enrichment analysis of genes at the *QFe/Se.yaas-2D***.** The abscissa is –*log*_10_ (FDR). FDR, False discovery rate, reflects the probability of a false positive rate in the test.


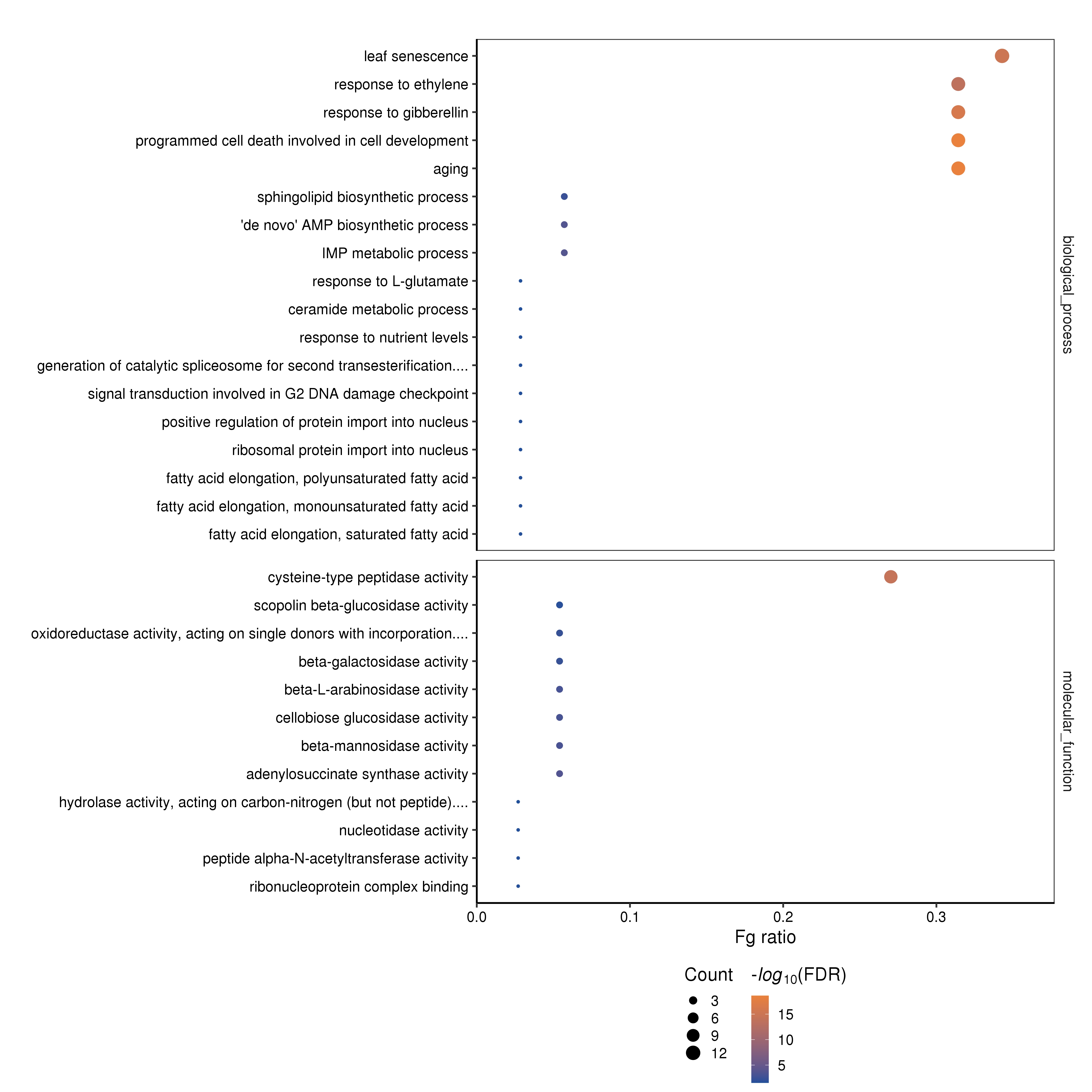


**Supplementary Figure 2** Bubble plots of biological processes and molecular function of GO enrichment analysis of genes at the *QMn/Zn.yaas-4D***.** The abscissa is –*log*_10_ (FDR). FDR, False discovery rate, reflects the probability of a false positive rate in the test.
