# Supplementary material for: Molecular mapping and validation of quantitative trait loci for content of micronutrients in wheat grain
Source: Front Plant Sci. 2025 Jan 17;15:1522465. doi: 10.3389/fpls.2024.1522465 (PMC11782267; doi:10.3389/fpls.2024.1522465)
Supplement: Supplementary file 2 [file DataSheet2.docx]

**Supplementary Table 1** Content of micronutrients and genotypes of the one hundred and forty-nine advanced lines.

|  | **Phenotype mg/kg** | | | | | **Genotype** | | | | | |
| --- | --- | --- | --- | --- | --- | --- | --- | --- | --- | --- | --- |
| **Advanced line** | **Mean-Mn** | **Mean-Fe** | **Mean-Cu** | **Mean-Zn** | **Mean-Se** | **KASP.1A** | **KASP.1B** | **KASP.2D** | **KASP.4D** | **KASP.7A** | **KASP.7D** |
| Line 1 | 16.56 | 38.12 | 5.71 | 77.45 | 0.03 | B | B | B | A | A | A |
| Line 2 | 17.09 | 44.57 | 5.89 | 88.83 | 0.05 | B | B | B | A | B | B |
| Line 3 | 6.04 | 35.90 | 5.83 | 55.18 | 0.06 | B | B | A | A | B | A |
| Line 4 | 6.02 | 29.93 | 6.83 | 58.39 | 0.03 | A | B | B | A | A | B |
| Line 5 | 6.20 | 25.06 | 5.62 | 50.21 | 0.06 | B | B | B | B | A | B |
| Line 6 | 6.56 | 35.6 | 3.25 | 36.85 | 0.09 | B | B | B | A | A | A |
| Line 7 | 16.19 | 35.55 | 5.89 | 77.76 | 0.03 | B | B | A | A | B | B |
| Line 8 | 5.31 | 20.97 | 4.81 | 46.99 | 0.06 | A | A | B | A | B | B |
| Line 9 | 18.89 | 42.16 | 7.47 | 79.55 | 0.07 | B | B | A | A | A | B |
| Line 10 | 5.78 | 28.18 | 3.47 | 41.53 | 0.07 | A | A | B | A | B | A |
| Line 11 | 7.93 | 20.30 | 3.05 | 50.17 | 0.09 | B | A | B | A | B | A |
| Line 12 | 7.38 | 47.97 | 4.55 | 80.89 | 0.08 | B | A | B | A | B | B |
| Line 13 | 13.77 | 31.13 | 3.50 | 59.55 | 0.08 | B | B | A | A | B | B |
| Line 14 | 14.10 | 31.00 | 7.20 | 81.04 | 0.04 | B | B | B | A | A | B |
| Line 15 | 12.17 | 38.58 | 9.64 | 118.66 | 0.07 | B | B | B | A | A | B |
| Line 16 | 13.71 | 28.64 | 7.31 | 90.61 | 0.06 | B | B | B | A | B | B |
| Line 17 | 6.40 | 26.27 | 5.15 | 35.41 | 0.07 | A | A | A | A | A | A |
| Line 18 | 6.56 | 23.23 | 3.93 | 53.24 | 0.03 | A | A | B | A | B | B |
| Line 19 | 21.28 | 60.08 | 8.47 | 116.29 | 0.12 | B | B | A | A | A | B |
| Line 20 | 7.56 | 29.45 | 5.35 | 33.07 | 0.06 | B | B | B | A | B | B |
| Line 21 | 9.11 | 33.89 | 3.46 | 40.14 | 0.03 | B | B | A | A | A | B |
| Line 22 | 6.20 | 46.34 | 6.56 | 63.61 | 0.08 | B | A | A | A | B | A |
| Line 23 | 7.14 | 31.68 | 4.99 | 32.03 | 0.04 | A | A | B | B | A | A |
| Line 24 | 9.61 | 32.56 | 7.85 | 55.38 | 0.07 | B | B | A | A | A | B |
| Line 25 | 13.87 | 68.51 | 5.91 | 66.33 | 0.10 | B | B | A | A | A | A |
| Line 26 | 7.34 | 47.21 | 4.41 | 48.23 | 0.03 | B | A | B | B | B | A |
| Line 27 | 2.97 | 51.93 | 2.71 | 35.58 | 0.08 | A | B | A | B | B | A |
| Line 28 | 6.22 | 45.95 | 6.65 | 44.37 | 0.07 | A | A | A | A | A | A |
| Line 29 | 5.29 | 40.54 | 4.46 | 44.93 | 0.03 | B | B | B | A | A | A |
| Line 30 | 7.65 | 37.64 | 3.32 | 42.2 | 0.08 | B | B | A | A | B | B |
| Line 31 | 5.73 | 23.95 | 2.77 | 83.58 | 0.14 | A | B | B | A | B | B |
| Line 32 | 6.98 | 27.05 | 2.31 | 33.44 | 0.07 | A | A | B | A | B | A |
| Line 33 | 23.01 | 66.24 | 8.61 | 135.64 | 0.08 | B | B | A | A | B | B |
| Line 34 | 8.22 | 46.70 | 5.07 | 51.10 | 0.03 | B | B | A | A | A | A |
| Line 35 | 4.75 | 52.26 | 4.4 | 58.89 | 0.05 | B | B | B | A | A | B |
| Line 36 | 12.36 | 78.91 | 5.7 | 85.94 | 0.07 | A | A | A | A | A | B |
| Line 37 | 18.06 | 69.28 | 9.15 | 106.36 | 0.06 | B | B | A | A | A | B |
| Line 38 | 10.10 | 59.56 | 3.05 | 42.03 | 0.05 | A | B | A | A | A | A |
| Line 39 | 13.91 | 47.90 | 2.07 | 47.43 | 0.06 | B | B | B | A | B | A |
| Line 40 | 18.98 | 51.71 | 6.24 | 64.32 | 0.07 | A | B | A | A | A | A |
| Line 41 | 4.59 | 49.92 | 4.88 | 59.20 | 0.09 | A | A | B | B | A | B |
| Line 42 | 7.97 | 50.74 | 6.22 | 71.18 | 0.09 | B | B | A | B | B | B |
| Line 43 | 8.43 | 47.74 | 5.57 | 93.31 | 0.07 | B | B | A | A | A | A |
| Line 44 | 4.62 | 37.63 | 4.81 | 41.98 | 0.09 | B | B | B | B | A | A |
| Line 45 | 9.48 | 37.24 | 3.32 | 53.01 | 0.04 | B | B | A | B | A | A |
| Line 46 | 4.18 | 36.21 | 3.19 | 36.34 | 0.03 | B | B | A | B | A | A |
| Line 47 | 8.21 | 51.38 | 4.68 | 66.15 | 0.03 | B | B | B | A | A | B |
| Line 48 | 6.13 | 36.19 | 3.23 | 48.56 | 0.05 | A | A | B | A | A | A |
| Line 49 | 9.85 | 53.83 | 6.04 | 44.32 | 0.02 | B | A | A | A | A | B |
| Line 50 | 26.16 | 48.53 | 5.79 | 65.26 | 0.07 | B | B | A | B | B | B |
| Line 51 | 11.89 | 24.20 | 5.20 | 59.05 | 0.13 | B | B | A | A | B | A |
| Line 52 | 7.64 | 14.99 | 3.04 | 34.42 | 0.03 | A | A | B | A | A | B |
| Line 53 | 5.88 | 14.81 | 3.32 | 36.96 | 0.02 | A | A | A | A | A | A |
| Line 54 | 6.23 | 19.02 | 4.87 | 40.92 | 0.02 | A | A | B | A | A | A |
| Line 55 | 12.17 | 53.12 | 6.59 | 52.13 | 0.07 | B | B | A | B | A | A |
| Line 56 | 8.66 | 30.24 | 2.70 | 50.27 | 0.06 | B | B | B | A | B | B |
| Line 57 | 7.04 | 36.85 | 7.04 | 53.77 | 0.09 | B | B | A | A | A | B |
| Line 58 | 10.34 | 32.10 | 3.22 | 45.76 | 0.03 | B | B | B | A | B | A |
| Line 59 | 4.92 | 15.44 | 3.66 | 46.21 | 0.01 | B | A | B | A | A | A |
| Line 60 | 8.21 | 21.44 | 3.33 | 71.44 | 0.02 | B | B | B | A | B | A |
| Line 61 | 7.47 | 19.50 | 2.75 | 60.39 | 0.01 | A | A | B | A | A | A |
| Line 62 | 9.18 | 14.00 | 3.99 | 64.23 | 0.02 | B | B | B | A | B | A |
| Line 63 | 5.93 | 23.33 | 2.77 | 47.37 | 0.02 | A | A | B | A | B | A |
| Line 64 | 7.34 | 13.00 | 2.17 | 68.92 | 0.09 | A | A | A | B | B | B |
| Line 65 | 3.99 | 15.80 | 2.77 | 74.94 | 0.14 | A | A | B | A | A | A |
| Line 66 | 6.64 | 26.73 | 4.67 | 68.41 | 0.02 | B | B | B | A | B | A |
| Line 67 | 9.71 | 15.43 | 5.16 | 76.00 | 0.03 | B | A | B | A | A | A |
| Line 68 | 10.59 | 15.98 | 3.94 | 45.61 | 0.01 | B | B | B | A | A | A |
| Line 69 | 9.97 | 17.25 | 2.65 | 41.42 | 0.03 | B | B | B | A | B | A |
| Line 70 | 8.63 | 18.85 | 4.63 | 44.43 | 0.07 | B | B | A | A | B | A |
| Line 71 | 6.62 | 15.77 | 1.88 | 46.33 | 0.01 | A | A | B | A | B | B |
| Line 72 | 5.64 | 15.17 | 2.92 | 79.85 | 0.06 | A | A | A | A | A | B |
| Line 73 | 8.55 | 25.11 | 3.74 | 49.07 | 0.02 | B | B | B | B | B | B |
| Line 74 | 7.99 | 20.48 | 3.19 | 40.45 | 0.06 | A | A | A | B | B | B |
| Line 75 | 6.83 | 30.63 | 8.40 | 46.38 | 0.01 | B | B | B | B | A | B |
| Line 76 | 7.80 | 16.96 | 5.89 | 41.53 | 0.09 | B | B | A | B | A | B |
| Line 77 | 5.99 | 20.24 | 4.75 | 70.63 | 0.03 | A | A | B | A | A | A |
| Line 78 | 6.82 | 18.23 | 4.55 | 35.06 | 0.02 | B | B | B | B | B | A |
| Line 79 | 9.09 | 15.81 | 3.73 | 38.97 | 0.02 | B | B | B | B | B | A |
| Line 80 | 5.88 | 23.11 | 1.35 | 41.56 | 0.07 | B | B | A | B | B | A |
| Line 81 | 8.05 | 22.07 | 4.80 | 49.15 | 0.01 | B | A | B | A | B | A |
| Line 82 | 6.38 | 11.50 | 3.25 | 51.25 | 0.06 | B | A | A | B | B | A |
| Line 83 | 8.86 | 25.37 | 3.26 | 55.48 | 0.07 | A | B | B | A | B | B |
| Line 84 | 5.90 | 21.74 | 1.63 | 34.65 | 0.05 | B | B | A | B | B | A |
| Line 85 | 5.63 | 30.29 | 5.01 | 41.04 | 0.13 | A | A | A | A | A | A |
| Line 86 | 5.72 | 33.37 | 4.14 | 37.52 | 0.09 | B | B | A | A | A | B |
| Line 87 | 7.36 | 16.91 | 2.50 | 39.09 | 0.07 | B | B | A | B | B | A |
| Line 88 | 7.52 | 33.18 | 3.44 | 44.65 | 0.05 | B | B | B | A | B | A |
| Line 89 | 5.45 | 22.67 | 0.74 | 57.04 | 0.03 | A | A | B | A | B | A |
| Line 90 | 5.82 | 26.20 | 2.17 | 36.63 | 0.09 | A | A | B | A | A | A |
| Line 91 | 4.35 | 31.75 | 1.17 | 57.78 | 0.04 | B | B | B | B | B | A |
| Line 92 | 6.06 | 21.21 | 3.61 | 43.37 | 0.07 | B | B | B | A | B | A |
| Line 93 | 9.19 | 17.91 | 2.64 | 63.19 | 0.06 | B | B | B | A | A | B |
| Line 94 | 5.62 | 13.39 | 1.88 | 53.84 | 0.09 | A | B | A | A | B | B |
| Line 95 | 7.18 | 29.01 | 4.13 | 87.05 | 0.08 | B | B | B | A | B | A |
| Line 96 | 5.54 | 17.86 | 1.29 | 33.96 | 0.07 | A | A | B | A | A | A |
| Line 97 | 5.57 | 32.77 | 2.31 | 37.24 | 0.07 | B | B | B | B | A | B |
| Line 98 | 4.85 | 18.82 | 0.85 | 34.64 | 0.12 | A | B | B | A | B | A |
| Line 99 | 5.66 | 16.32 | 1.43 | 46.16 | 0.09 | A | A | A | A | A | B |
| Line 100 | 8.86 | 43.66 | 3.53 | 65.68 | 0.05 | B | B | A | A | B | A |
| Line 101 | 4.47 | 24.60 | 4.47 | 75.39 | 0.05 | B | B | B | A | B | B |
| Line 102 | 6.26 | 31.89 | 3.45 | 47.49 | 0.07 | B | B | B | A | A | B |
| Line 103 | 11.07 | 20.35 | 1.38 | 48.19 | 0.03 | B | B | B | A | B | A |
| Line 104 | 16.22 | 24.87 | 4.34 | 59.53 | 0.02 | B | B | A | A | A | B |
| Line 105 | 4.20 | 26.54 | 3.15 | 49.26 | 0.07 | B | A | A | B | A | B |
| Line 106 | 4.80 | 20.84 | 0.95 | 41.09 | 0.05 | A | A | B | A | A | A |
| Line 107 | 4.48 | 41.10 | 0.71 | 61.44 | 0.07 | A | A | A | A | B | A |
| Line 108 | 6.75 | 44.59 | 1.56 | 63.02 | 0.07 | A | A | A | A | B | B |
| Line 109 | 4.68 | 18.00 | 0.02 | 69.57 | 0.07 | A | A | B | A | A | B |
| Line 110 | 4.57 | 18.65 | 2.03 | 38.86 | 0.05 | B | A | B | B | B | A |
| Line 111 | 8.51 | 25.30 | 0.88 | 72.53 | 0.08 | A | A | B | A | B | A |
| Line 112 | 5.03 | 16.36 | 3.39 | 77.04 | 0.08 | A | A | B | A | A | B |
| Line 113 | 4.35 | 36.78 | 0.58 | 85.3 | 0.05 | A | B | B | B | A | B |
| Line 114 | 9.87 | 35.32 | 1.96 | 79.39 | 0.04 | B | B | B | A | B | B |
| Line 115 | 11.00 | 43.12 | 1.28 | 107.35 | 0.05 | A | A | A | A | B | B |
| Line 116 | 10.09 | 37.52 | 3.94 | 54.86 | 0.05 | B | B | B | A | B | B |
| Line 117 | 7.68 | 45.07 | 2.96 | 43.15 | 0.03 | A | A | B | A | B | A |
| Line 118 | 19.46 | 42.62 | 6.77 | 77.88 | 0.06 | B | A | A | A | A | A |
| Line 119 | 16.87 | 15.03 | 5.21 | 40.25 | 0.02 | A | A | A | A | A | B |
| Line 120 | 5.36 | 14.24 | 2.10 | 48.60 | 0.07 | A | A | A | A | A | A |
| Line 121 | 7.96 | 20.37 | 2.19 | 40.27 | 0.04 | A | B | B | A | A | A |
| Line 122 | 6.99 | 15.50 | 2.43 | 42.77 | 0.07 | A | A | B | A | A | A |
| Line 123 | 5.82 | 14.82 | 9.44 | 65.48 | 0.06 | A | B | A | B | A | B |
| Line 124 | 11.11 | 45.19 | 3.39 | 67.35 | 0.05 | B | B | A | A | B | A |
| Line 125 | 4.68 | 20.29 | 4.40 | 40.75 | 0.07 | A | A | A | A | A | A |
| Line 126 | 9.47 | 29.49 | 3.93 | 67.46 | 0.06 | B | A | A | A | B | B |
| Line 127 | 10.11 | 23.07 | 2.37 | 46.6 | 0.05 | A | B | B | A | B | A |
| Line 128 | 16.16 | 15.84 | 2.30 | 60.77 | 0.04 | B | B | B | A | B | A |
| Line 129 | 5.22 | 25.14 | 2.70 | 41.97 | 0.04 | B | B | B | A | A | B |
| Line 130 | 5.98 | 26.30 | 4.34 | 42.54 | 0.07 | A | A | A | A | A | B |
| Line 131 | 6.36 | 17.59 | 4.31 | 48.88 | 0.08 | A | B | A | A | B | A |
| Line 132 | 5.96 | 33.22 | 3.65 | 45.49 | 0.03 | A | B | A | A | B | A |
| Line 133 | 5.22 | 15.10 | 1.01 | 40.92 | 0.04 | A | B | A | A | B | A |
| Line 134 | 5.28 | 18.49 | 2.03 | 48.49 | 0.05 | A | A | B | A | B | B |
| Line 135 | 3.38 | 24.46 | 2.33 | 59.63 | 0.07 | A | A | B | A | A | A |
| Line 136 | 6.60 | 15.49 | 2.32 | 46.16 | 0.13 | A | A | A | A | B | B |
| Line 137 | 5.40 | 28.36 | 3.03 | 38.65 | 0.11 | A | A | A | B | A | A |
| Line 138 | 4.35 | 14.76 | 1.35 | 33.34 | 0.02 | A | A | B | B | A | A |
| Line 139 | 25.34 | 22.43 | 6.64 | 39.25 | 0.03 | A | B | B | B | B | B |
| Line 140 | 3.90 | 20.17 | 1.95 | 36.51 | 0.01 | A | A | A | A | B | B |
| Line 141 | 4.70 | 18.88 | 4.07 | 42.09 | 0.03 | B | A | B | B | B | B |
| Line 142 | 5.68 | 16.14 | 1.47 | 43.47 | 0.03 | A | A | B | A | B | B |
| Line 143 | 7.35 | 22.53 | 2.31 | 44.20 | 0.05 | A | B | B | A | B | A |
| Line 144 | 4.74 | 25.95 | 2.98 | 39.46 | 0.04 | A | A | B | B | A | A |
| Line 145 | 5.26 | 16.46 | 3.91 | 35.10 | 0.10 | A | A | B | B | A | A |
| Line 146 | 18.14 | 22.48 | 5.55 | 76.92 | 0.12 | B | B | A | A | B | B |
| Line 147 | 5.01 | 20.88 | 3.17 | 51.99 | 0.12 | A | A | A | A | A | A |
| Line 148 | 11.36 | 22.76 | 3.37 | 55.80 | 0.05 | A | A | B | A | B | A |
| Line 149 | 16.85 | 17.57 | 5.07 | 62.38 | 0.04 | A | A | B | A | B | A |

A indicates Yangmai 4 allele; B indicates Yanzhan 1 allele.

**Supplementary Table 2** The list of the annotated high-confidence genes within the genomic region of *QFe/Se.yaas-2D.*

| **Gene ID^a^** | **Location^b^ (Mb)** | | **Description^c^** | ***Oryza sativa*^d^** | ***Arabidopsis thaliana*^e^** |
| --- | --- | --- | --- | --- | --- |
| *TraesCS2D03G0955600* | 536.48 | Cytochrome P450 99A2 [UniProtKB/Swiss-Prot:Q7X7X4] | | *Os04g0180400* | *AT4G13770* |
| *TraesCS2D03G0955700* | 536.51 | Ent-kaur-16-ene synthase, chloroplastic [UniProtKB/Swiss-Prot:Q0JA82] | | *Os04g0611800* | *AT1G79460* |
| *TraesCS2D03G0955800* | 536.53 | Acyclic sesquiterpene synthase [UniProtKB/Swiss-Prot:Q84ZW8] | | *Os04g0612000* | *AT1G79460* |
| *TraesCS2D03G0956000* | 536.90 | Acyclic sesquiterpene synthase [UniProtKB/Swiss-Prot:Q84ZW8] | | *Os04g0612000* | *AT1G79460* |
| *TraesCS2D03G0956100* | 536.91 | Multiple RNA-binding domain-containing protein 1 [UniProtKB/Swiss-Prot:Q5AJS6] | | *Os04g0611500* | *AT4G13850* |
| *TraesCS2D03G0956200* | 536.92 | Vacuolar-sorting receptor 7 [UniProtKB/Swiss-Prot:Q8L7E3] | | *Os04g0611400* | *AT2G34940* |
| *TraesCS2D03G0956400* | 536.92 | NA | | *Os04g0611300* | NA |
| *TraesCS2D03G0956500* | 536.99 | Cationic amino acid transporter 8, vacuolar [UniProtKB/Swiss-Prot:Q9SHH0] | | *Os06g0539400* | *AT2G34960* |
| *TraesCS2D03G0957400* | 537.19 | Protein ECERIFERUM 26-like [UniProtKB/Swiss-Prot:Q9LIS1] | | *Os04g0611200* | *AT4G13840* |
| *TraesCS2D03G0957600* | 537.21 | 26S proteasome non-ATPase regulatory subunit 8 homolog A [UniProtKB/Swiss-Prot:Q9SGW3] | | *Os07g0435100* | *AT1G64520* |
| *TraesCS2D03G0957700* | 537.22 | Serine/threonine-protein kinase CTR1 [UniProtKB/Swiss-Prot:Q05609] | | *Os04g0610900* | NA |
| *TraesCS2D03G0957800* | 537.32 | MEIOTIC F-BOX protein MOF [UniProtKB/Swiss-Prot:Q7X7A4] | | NA | *AT4G14096* |
| *TraesCS2D03G0957900* | 537.33 | UPF0481 protein At3g47200 [UniProtKB/Swiss-Prot:Q9SD53] | | NA | NA |
| *TraesCS2D03G0958100* | 537.34 | UPF0481 protein At3g47200 [UniProtKB/Swiss-Prot:Q9SD53] | | NA | NA |
| *TraesCS2D03G0958500* | 537.45 | NA | | *Os11g0496500* | *AT3G27030* |
| *TraesCS2D03G0958800* | 537.46 | NA | | *Os11g0496500* | *AT3G48660* |
| *TraesCS2D03G0958900* | 537.46 | Oxygen-dependent coproporphyrinogen-III oxidase, chloroplastic [UniProtKB/Swiss-Prot:Q42840] | | *Os04g0610800* | *AT1G03475* |
| *TraesCS2D03G0959000* | 537.50 | Probable potassium transporter 15 [UniProtKB/Swiss-Prot:Q7XPL3] | | *Os04g0610700* | *AT5G09400* |
| *TraesCS2D03G0959100* | 537.52 | NA | | *Os04g0610600* | NA |
| *TraesCS2D03G0959600* | 537.70 | NA | | *Os04g0610600* | NA |
| *TraesCS2D03G0959700* | 537.72 | Uncharacterized protein ECU03_1610 [UniProtKB/Swiss-Prot:Q8SVY9] | | *Os04g0610600* | NA |
| *TraesCS2D03G0959800* | 537.93 | Probable metal-nicotianamine transporter YSL7 [UniProtKB/Swiss-Prot:Q6ZGM7] | | NA | NA |
| *TraesCS2D03G0960100* | 537.93 | Methionine aminopeptidase 1B, chloroplastic [UniProtKB/Swiss-Prot:Q9FV52] | | *Os04g0610500* | *AT1G13270* |
| *TraesCS2D03G0960200* | 538.05 | Ethylene-responsive transcription factor 4 [UniProtKB/Swiss-Prot:O80340] | | *Os04g0610400* | *AT4G13620* |
| *TraesCS2D03G0960400* | 538.27 | G-type lectin S-receptor-like serine/threonine-protein kinase At1g34300 [UniProtKB/Swiss-Prot:Q9XID3] | | *Os04g0616200* | NA |
| *TraesCS2D03G0960500* | 538.28 | Protein FORGETTER 1 [UniProtKB/Swiss-Prot:F4IF36] | | *Os04g0610000* | NA |
| *TraesCS2D03G0960600* | 538.31 | Protein FORGETTER 1 [UniProtKB/Swiss-Prot:F4IF36] | | *Os04g0609900* | NA |
| *TraesCS2D03G0960800* | 538.59 | Protein FORGETTER 1 [UniProtKB/Swiss-Prot:F4IF36] | | *Os04g0609900* | NA |
| *TraesCS2D03G0960900* | 538.60 | Trimethyltridecatetraene synthase [UniProtKB/Swiss-Prot:A0A1D6F9Y9] | | *Os09g0441400* | *AT4G13770* |
| *TraesCS2D03G0961000* | 538.66 | Protein FORGETTER 1 [UniProtKB/Swiss-Prot:F4IF36] | | *Os04g0609700* | *AT1G79350* |
| *TraesCS2D03G0961200* | 538.73 | Organic cation/carnitine transporter 7 [UniProtKB/Swiss-Prot:Q940M4] | | *Os04g0609200* | *AT3G13050* |
| *TraesCS2D03G0961400* | 538.80 | Probable protein phosphatase 2C 44 [UniProtKB/Swiss-Prot:Q0JAA0] | | *Os04g0609600* | *AT4G28400* |
| *TraesCS2D03G0962200* | 538.99 | Pumilio homolog 1 [UniProtKB/Swiss-Prot:Q9ZW07] | | NA | NA |
| *TraesCS2D03G0962700* | 539.17 | Shikimate O-hydroxycinnamoyltransferase [UniProtKB/Swiss-Prot:Q8GSM7] | | *Os04g0609500* | NA |
| *TraesCS2D03G0962900* | 539.22 | 50S ribosomal protein L22 [UniProtKB/Swiss-Prot:B2IK67] | | *Os01g0616500* | NA |
| *TraesCS2D03G0963000* | 539.22 | Hydroxycinnamoyltransferase 2 [UniProtKB/Swiss-Prot:Q6K638] | | *Os04g0609300* | *AT4G13840* |
| *TraesCS2D03G0963100* | 539.26 | G-type lectin S-receptor-like serine/threonine-protein kinase SD2-5 [UniProtKB/Swiss-Prot:Q8RWZ5] | | *Os04g0616600* | NA |
| *TraesCS2D03G0963400* | 539.41 | Ent-kaurene oxidase 2 [UniProtKB/Swiss-Prot:Q5Z5R4] | | *Os06g0570100* | *AT4G13770* |
| *TraesCS2D03G0963500* | 539.42 | NA | | NA | NA |
| *TraesCS2D03G0963600* | 539.43 | Pre-mRNA-splicing factor CWC21 [UniProtKB/Swiss-Prot:Q4IB70] | | *Os04g0609100* | NA |
| *TraesCS2D03G0963800* | 539.44 | DNA-directed RNA polymerase II subunit RPB1 [UniProtKB/Swiss-Prot:P18616] | | *Os05g0151000* | *AT4G35800* |
| *TraesCS2D03G0964200* | 539.87 | 26S proteasome non-ATPase regulatory subunit 1 homolog A [UniProtKB/Swiss-Prot:O48844] | | *Os04g0608500* | *AT2G32730* |
| *TraesCS2D03G0964400* | 539.88 | Serine/threonine-protein kinase STY13 [UniProtKB/Swiss-Prot:Q9ZQ31] | | *Os04g0608900* | *AT3G22750* |
| *TraesCS2D03G0964600* | 539.88 | Nuclear pore complex protein NUP88 [UniProtKB/Swiss-Prot:Q9FFK6] | | *Os04g0608400* | NA |
| *TraesCS2D03G0964700* | 539.90 | Auxin-responsive protein SAUR71 [UniProtKB/Swiss-Prot:Q9SGU2] | | *Os04g0608300* | *AT4G31320* |
| *TraesCS2D03G0964900* | 540.00 | Galacturonokinase [UniProtKB/Swiss-Prot:Q8VYG2] | | *Os04g0608100* | *AT3G10700* |
| *TraesCS2D03G0965100* | 540.06 | Probable cation transporter HKT7 [UniProtKB/Swiss-Prot:Q7XPF7] | | *Os04g0607600* | NA |
| *TraesCS2D03G0965200* | 540.16 | Probable cation transporter HKT7 [UniProtKB/Swiss-Prot:Q7XPF7] | | *Os04g0607600* | NA |
| *TraesCS2D03G0965400* | 540.19 | Probable cation transporter HKT7 [UniProtKB/Swiss-Prot:Q7XPF7] | | *Os04g0607600* | NA |
| *TraesCS2D03G0965500* | 540.20 | Cation transporter HKT4 [UniProtKB/Swiss-Prot:Q7XPF8] | | *Os04g0607500* | NA |
| *TraesCS2D03G0966000* | 540.64 | NA | | *Os04g0607150* | *AT4G31340* |
| *TraesCS2D03G0966100* | 540.67 | NA | | NA | NA |
| *TraesCS2D03G0966200* | 540.82 | Glycosyltransferase BC10 [UniProtKB/Swiss-Prot:Q65XS5] | | *Os04g0607100* | *AT4G31350* |
| *TraesCS2D03G0966300* | 540.82 | Probable plastid-lipid-associated protein 8, chloroplastic [UniProtKB/Swiss-Prot:Q941D3] | | *Os04g0607000* | *AT5G19940* |
| *TraesCS2D03G0966400* | 540.83 | Probable leucine-rich repeat receptor-like protein kinase At5g49770 [UniProtKB/Swiss-Prot:Q9LT96] | | *Os04g0616200* | *AT4G29990* |
| *TraesCS2D03G0966500* | 540.84 | Putative RNA methyltransferase At5g10620 [UniProtKB/Swiss-Prot:Q9LXB4] | | *Os04g0606900* | *AT5G10620* |
| *TraesCS2D03G0966700* | 540.95 | Chalcone synthase 2 [UniProtKB/Swiss-Prot:P53415] | | *Os11g0530600* | *AT5G13930* |
| *TraesCS2D03G0966900* | 540.95 | NA | | NA | NA |
| *TraesCS2D03G0967300* | 540.96 | NA | | NA | NA |
| *TraesCS2D03G0967800* | 541.07 | NA | | *Os04g0606700* | NA |

^a^Gene ID (*Triticum aestivum*), *T. aestivum* gene transcripts and their domains that were available in Ensembl (using the transcript table link). ^b^Physical location (Mb), the physical location is based on the Chinese Spring 2.1 reference genome (RefSeq v2.1). ^c^Annotation, the description of the corresponding gene, NA represents the unknown function; ^d^Orthologs gene ID in Rice; ^e^Orthologs gene ID in Arabidopsis thaliana.

**Supplementary Table 3** The list of the annotated high-confidence genes within the genomic region of *QMn/Zn.yaas-4D.*

| **Gene ID^a^** | **Location^b^ (Mb)** | **Description^c^** | ***Oryza sativa^d^*** | ***Arabidopsis thaliana^e^*** |
| --- | --- | --- | --- | --- |
| *TraesCS4D03G0057000* | 15.59 | Protein yippee-like [UniProtKB/Swiss-Prot:P59234] | *Os03g0698500* | *AT2G40110* |
| *TraesCS4D03G0057300* | 15.71 | Zinc finger CCCH domain-containing protein 24 [UniProtKB/Swiss-Prot:Q10EL1] | *Os03g0698800* | *NA* |
| *TraesCS4D03G0057400* | 15.72 | Alkaline ceramidase [UniProtKB/Swiss-Prot:Q94IB9] | *Os03g0698900* | *AT4G22330* |
| *TraesCS4D03G0057700* | 15.77 | Denticleless protein homolog [UniProtKB/Swiss-Prot:Q5ZJW8] | *Os03g0699100* | NA |
| *TraesCS4D03G0057800* | 15.78 | Pescadillo homolog [UniProtKB/Swiss-Prot:Q851S7] | *Os03g0699200* | *AT5G14520* |
| *TraesCS4D03G0057900* | 15.79 | Adenylosuccinate synthetase 2, chloroplastic [UniProtKB/Swiss-Prot:C5WNV2] | *Os03g0699300* | *AT3G57610* |
| *TraesCS4D03G0058000* | 15.80 | Adenylosuccinate synthetase 2, chloroplastic [UniProtKB/Swiss-Prot:Q851S8] | *Os03g0699300* | *AT3G57610* |
| *TraesCS4D03G0058100* | 15.80 | N-terminal acetyltransferase B complex catalytic subunit NAA20 [UniProtKB/Swiss-Prot:Q8LGI8] | *Os03g0699400* | *AT1G03150* |
| *TraesCS4D03G0058500* | 15.86 | Multicopper oxidase LPR1 homolog 1 [UniProtKB/Swiss-Prot:Q9AWU4] | *Os01g0126100* | *AT1G23010* |
| *TraesCS4D03G0058600* | 15.87 | Probable linoleate 9S-lipoxygenase 4 [UniProtKB/Swiss-Prot:Q53RB0] | *Os03g0700700* | *AT1G55020* |
| *TraesCS4D03G0058900* | 15.91 | Linoleate 9S-lipoxygenase 1 [UniProtKB/Swiss-Prot:P29114] | *Os03g0700400* | *AT1G55020* |
| *TraesCS4D03G0059200* | 15.93 | F-box protein At1g55000 [UniProtKB/Swiss-Prot:Q9FZ32] | *Os03g0699600* | *AT1G55000* |
| *TraesCS4D03G0059400* | 15.96 | NA | NA | NA |
| *TraesCS4D03G0059600* | 15.96 | Pentatricopeptide repeat-containing protein At2g13600 [UniProtKB/Swiss-Prot:Q9SIT7] | *Os03g0701400* | NA |
| *TraesCS4D03G0059700* | 16.01 | NA | *Os03g0701300* | NA |
| *TraesCS4D03G0059800* | 16.02 | Uncharacterized protein C24B11.05 [UniProtKB/Swiss-Prot:Q09893] | *Os03g0701200* | *AT2G32150* |
| *TraesCS4D03G0059900* | 16.02 | Pre-mRNA-splicing factor 18 [UniProtKB/Swiss-Prot:Q8BM39] | *Os03g0701100* | NA |
| *TraesCS4D03G0060000* | 16.05 | Importin-5 [UniProtKB/Swiss-Prot:Q8BKC5] | *Os03g0701000* | *AT5G19820* |
| *TraesCS4D03G0060200* | 16.10 | Protein ETHYLENE-INSENSITIVE 2 [UniProtKB/Swiss-Prot:Q0D8I9] | *Os03g0700800* | NA |
| *TraesCS4D03G0060300* | 16.15 | Elongation of fatty acids protein 3-like [UniProtKB/Swiss-Prot:Q9SYY4] | *Os03g0701500* | NA |
| *TraesCS4D03G0060400* | 16.15 | NA | *Os03g0701600* | NA |
| *TraesCS4D03G0060500* | 16.15 | NA | NA | NA |
| *TraesCS4D03G0060800* | 16.23 | Probable ethylene response sensor 1 [UniProtKB/Swiss-Prot:Q53RH0] | *Os03g0701700* | *AT1G66340* |
| *TraesCS4D03G0061100* | 16.24 | Phosphatidylinositol 4-phosphate 5-kinase 1 [UniProtKB/Swiss-Prot:Q6EX42] | *Os03g0701800* | NA |
| *TraesCS4D03G0061200* | 16.25 | Senescence-specific cysteine protease SAG39 [UniProtKB/Swiss-Prot:A2XQE8] | *Os04g0206300* | NA |
| *TraesCS4D03G0061300* | 16.26 | Senescence-specific cysteine protease SAG39 [UniProtKB/Swiss-Prot:A2XQE8] | *Os04g0207600* | *AT5G45890* |
| *TraesCS4D03G0061400* | 16.26 | Senescence-specific cysteine protease SAG39 [UniProtKB/Swiss-Prot:A2XQE8] | *Os04g0206300* | NA |
| *TraesCS4D03G0061500* | 16.26 | Senescence-specific cysteine protease SAG39 [UniProtKB/Swiss-Prot:A2XQE8] | *Os04g0207600* | *AT5G45890* |
| *TraesCS4D03G0061900* | 16.30 | Senescence-specific cysteine protease SAG39 [UniProtKB/Swiss-Prot:A2XQE8] | *Os04g0207600* | *AT5G45890* |
| *TraesCS4D03G0062000* | 16.31 | Senescence-specific cysteine protease SAG39 [UniProtKB/Swiss-Prot:A2XQE8] | *Os04g0207600* | *AT5G45890* |
| *TraesCS4D03G0062700* | 16.33 | Senescence-specific cysteine protease SAG39 [UniProtKB/Swiss-Prot:A2XQE8] | *Os04g0207600* | *AT5G45890* |
| *TraesCS4D03G0062800* | 16.33 | Senescence-specific cysteine protease SAG39 [UniProtKB/Swiss-Prot:Q7XWK5] | *Os04g0207600* | *AT5G45890* |
| *TraesCS4D03G0062900* | 16.33 | Senescence-specific cysteine protease SAG39 [UniProtKB/Swiss-Prot:A2XQE8] | *Os04g0207600* | NA |
| *TraesCS4D03G0063000* | 16.35 | Senescence-specific cysteine protease SAG39 [UniProtKB/Swiss-Prot:A2XQE8] | *Os04g0207600* | *AT5G45890* |
| *TraesCS4D03G0063100* | 16.35 | Senescence-specific cysteine protease SAG39 [UniProtKB/Swiss-Prot:A2XQE8] | *Os04g0206300* | *AT5G45890* |
| *TraesCS4D03G0063200* | 16.36 | NA | *Os03g0701900* | NA |
| *TraesCS4D03G0063300* | 16.37 | Putative UDP-rhamnose:rhamnosyltransferase 1 [UniProtKB/Swiss-Prot:Q66PF2] | *Os03g0702000* | NA |
| *TraesCS4D03G0063400* | 16.37 | UDP-glycosyltransferase 91C1 [UniProtKB/Swiss-Prot:Q9LTA3] | *Os03g0702500* | NA |
| *TraesCS4D03G0063600* | 16.43 | NA | *Os03g0702600* | NA |
| *TraesCS4D03G0063800* | 16.45 | Receptor-like protein 2 [UniProtKB/Swiss-Prot:Q9SHI3] | *Os03g0724300* | NA |
| *TraesCS4D03G0063900* | 16.53 | Probable sugar phosphate/phosphate translocator At3g17430 [UniProtKB/Swiss-Prot:Q9LRP2] | *Os03g0702700* | *AT3G17430* |
| *TraesCS4D03G0064000* | 16.54 | Eukaryotic peptide chain release factor subunit 1-3 [UniProtKB/Swiss-Prot:P35614] | *Os03g0702800* | *AT3G26618* |
| *TraesCS4D03G0064100* | 16.58 | NA | *Os03g0702900* | NA |
| *TraesCS4D03G0064200* | 16.59 | Beta-glucosidase 7 [UniProtKB/Swiss-Prot:Q75I93] | *Os03g0703000* | *AT3G18080* |
| *TraesCS4D03G0064300* | 16.61 | Beta-glucosidase 8 [UniProtKB/Swiss-Prot:Q75I94] | *Os03g0703100* | *AT3G18080* |
| *TraesCS4D03G0064400* | 16.64 | Zinc finger protein 593 homolog [UniProtKB/Swiss-Prot:Q9U239] | *Os08g0564500* | *AT2G36930* |
| *TraesCS4D03G0064500* | 16.65 | Protein OPAQUE10 [UniProtKB/Swiss-Prot:P0DKL2] | *Os03g0703300* | NA |
| *TraesCS4D03G0064600* | 16.66 | Mitogen-activated protein kinase kinase kinase 1 [UniProtKB/Swiss-Prot:Q39008] | *Os03g0703400* | NA |
| *TraesCS4D03G0065000* | 16.79 | Uncharacterized membrane protein At4g09580 [UniProtKB/Swiss-Prot:Q8L586] | *Os03g0703900* | *AT4G09580* |
| *TraesCS4D03G0065100* | 16.80 | 30S ribosomal protein S13, chloroplastic [UniProtKB/Swiss-Prot:P82163] | *Os03g0704000* | *AT5G14320* |
| *TraesCS4D03G0065300* | 16.80 | Probable plastid-lipid-associated protein 4, chloroplastic [UniProtKB/Swiss-Prot:Q9LU85] | *Os03g0704100* | *AT3G26070* |

^a^Gene ID (*Triticum aestivum*), *T. aestivum* gene transcripts and their domains that were available in Ensembl (using the transcript table link); ^b^Physical location (Mb), the physical location is based on the Chinese Spring 2.1 reference genome (RefSeq v2.1). ^c^Annotation, the description of the corresponding gene, NA represents the unknown function. ^d^Orthologs gene ID in Rice; ^e^Orthologs gene ID in Arabidopsis thaliana.

**Supplementary Table 4** Go enrichment analysis of genes at *QFe/Se.yaas-2D* and *QMn/Zn.yaas-4D*.

| QTL | Gene Id^a^ | Description | P-value | FDR^b^ | Number in foreground list |
| --- | --- | --- | --- | --- | --- |
| *QFe/Se.yaas-2D* | *TraesCS2D03G0960500/TraesCS2D03G0960600/TraesCS2D03G0960800/TraesCS2D03G0961000* | positive regulation of cellular response to heat | 1.08E-09 | 9.86E-08 | 4 |
|  | *TraesCS2D03G0960500/TraesCS2D03G0960600/TraesCS2D03G0960800/TraesCS2D03G0961000* | promoter-specific chromatin binding | 6.90E-09 | 2.83E-07 | 4 |
|  | *TraesCS2D03G0960500/TraesCS2D03G0960600/TraesCS2D03G0960800/TraesCS2D03G0961000* | nucleosome positioning | 1.49E-08 | 6.77E-07 | 4 |
|  | *TraesCS2D03G0960500/TraesCS2D03G0960600/TraesCS2D03G0960800/TraesCS2D03G0961000* | regulation of gene expression, epigenetic | 6.18E-08 | 1.88E-06 | 4 |
|  | *TraesCS2D03G0965100/TraesCS2D03G0965200/TraesCS2D03G0965400/TraesCS2D03G0965500* | cation transmembrane transporter activity | 1.59E-07 | 2.24E-06 | 4 |
|  | *TraesCS2D03G0955700/TraesCS2D03G0955800/TraesCS2D03G0956000* | ent-kaurene synthase activity | 2.04E-07 | 2.24E-06 | 3 |
|  | *TraesCS2D03G0960500/TraesCS2D03G0960600/TraesCS2D03G0960800/TraesCS2D03G0961000* | chromatin DNA binding | 2.19E-07 | 2.24E-06 | 4 |
|  | *TraesCS2D03G0960500/TraesCS2D03G0960600/TraesCS2D03G0960800/TraesCS2D03G0961000* | heat acclimation | 4.96E-07 | 1.13E-05 | 4 |
|  | *TraesCS2D03G0955800/TraesCS2D03G0956000* | (3R)-(E)-nerolidol synthase activity | 6.13E-06 | 4.19E-05 | 2 |
|  | *TraesCS2D03G0955800/TraesCS2D03G0956000* | farnesol biosynthetic process | 6.11E-06 | 6.96E-05 | 2 |
|  | *TraesCS2D03G0955800/TraesCS2D03G0956000* | sesquiterpenoid biosynthetic process | 6.11E-06 | 6.96E-05 | 2 |
|  | *TraesCS2D03G0955800/TraesCS2D03G0956000* | ent-kaurene biosynthetic process | 6.11E-06 | 6.96E-05 | 2 |
|  | *TraesCS2D03G0955800/TraesCS2D03G0956000* | geraniol biosynthetic process | 6.11E-06 | 6.96E-05 | 2 |
|  | *TraesCS2D03G0960500/TraesCS2D03G0960600/TraesCS2D03G0960800/TraesCS2D03G0961000* | histone binding | 1.76E-05 | 0.000103 | 4 |
|  | *TraesCS2D03G0955800/TraesCS2D03G0956000/TraesCS2D03G0957700/TraesCS2D03G0960200* | response to ethylene | 0.000208 | 0.00146 | 4 |
|  | *TraesCS2D03G0957700/TraesCS2D03G0960200* | negative regulation of ethylene-activated signaling pathway | 0.000433 | 0.00282 | 2 |
|  | *TraesCS2D03G0957700/TraesCS2D03G0964400* | protein serine/threonine/tyrosine kinase activity | 0.00542 | 0.0177 | 2 |
|  | *TraesCS2D03G0955600/TraesCS2D03G0960900/TraesCS2D03G0963400* | oxidoreductase activity, acting on paired donors, with incorporation or reduction of molecular oxygen, NAD(P)H as one donor, and incorporation of one atom of oxygen | 0.00632 | 0.0177 | 3 |
|  | *TraesCS2D03G0958900* | coproporphyrinogen oxidase activity | 0.00646 | 0.0177 | 1 |
|  | *TraesCS2D03G0963400* | ent-kaurene oxidase activity | 0.00646 | 0.0177 | 1 |
|  | *TraesCS2D03G0963400* | ent-kaur-16-en-19-ol oxidase activity | 0.00646 | 0.0177 | 1 |
|  | *TraesCS2D03G0963400* | ent-kaur-16-en-19-al oxidase activity | 0.00646 | 0.0177 | 1 |
|  | *TraesCS2D03G0955700/TraesCS2D03G0955800/TraesCS2D03G0956000* | magnesium ion binding | 0.00647 | 0.0177 | 3 |
|  | *TraesCS2D03G0964900* | galactose binding | 0.00775 | 0.0198 | 1 |
|  | *TraesCS2D03G0962700* | shikimate O-hydroxycinnamoyltransferase activity | 0.00839 | 0.0202 | 1 |
|  | *TraesCS2D03G0961200* | nicotinate transmembrane transporter activity | 0.0103 | 0.0223 | 1 |
|  | *TraesCS2D03G0961200* | N-methylnicotinate transmembrane transporter activity | 0.0103 | 0.0223 | 1 |
|  | *TraesCS2D03G0957400/TraesCS2D03G0963000* | transferase activity, transferring acyl groups other than amino-acyl groups | 0.0127 | 0.026 | 2 |
|  | *TraesCS2D03G0964600* | ribosomal small subunit export from nucleus | 0.00581 | 0.0353 | 1 |
|  | *TraesCS2D03G0963400* | ent-kaurene oxidation to kaurenoic acid | 0.00646 | 0.0367 | 1 |
|  | *TraesCS2D03G0964900* | carbohydrate phosphorylation | 0.0071 | 0.038 | 1 |
|  | *TraesCS2D03G0957700* | regulation of stem cell division | 0.00967 | 0.0447 | 1 |
|  | *TraesCS2D03G0957700* | regulation of post-embryonic root development | 0.00967 | 0.0447 | 1 |
|  | *TraesCS2D03G0961200* | nicotinate transport | 0.0103 | 0.0447 | 1 |
|  | *TraesCS2D03G0961200* | N-methylnicotinate transport | 0.0103 | 0.0447 | 1 |
|  | *TraesCS2D03G0964600* | protein export from nucleus | 0.011 | 0.0453 | 1 |
|  | *TraesCS2D03G0957600* | response to misfolded protein | 0.0116 | 0.0459 | 1 |
| *QMn/Zn.yaas-4D* | *TraesCS4D03G0061200/TraesCS4D03G0061300/TraesCS4D03G0061400/TraesCS4D03G0061500/TraesCS4D03G0061900/TraesCS4D03G0062000/TraesCS4D03G0062700/TraesCS4D03G0062800/TraesCS4D03G0062900/TraesCS4D03G0063000/TraesCS4D03G0063100* | senescence-associated vacuole | 3.89E-22 | 8.55E-21 | 11 |
|  | *TraesCS4D03G0061200/TraesCS4D03G0061300/TraesCS4D03G0061400/TraesCS4D03G0061500/TraesCS4D03G0061900/TraesCS4D03G0062000/TraesCS4D03G0062700/TraesCS4D03G0062800/TraesCS4D03G0062900/TraesCS4D03G0063000/TraesCS4D03G0063100* | aging | 2.96E-21 | 2.43E-19 | 11 |
|  | *TraesCS4D03G0061200/TraesCS4D03G0061300/TraesCS4D03G0061400/TraesCS4D03G0061500/TraesCS4D03G0061900/TraesCS4D03G0062000/TraesCS4D03G0062700/TraesCS4D03G0062800/TraesCS4D03G0062900/TraesCS4D03G0063000/TraesCS4D03G0063100* | programmed cell death involved in cell development | 6.10E-21 | 2.50E-19 | 11 |
|  | *TraesCS4D03G0061200/TraesCS4D03G0061300/TraesCS4D03G0061400/TraesCS4D03G0061500/TraesCS4D03G0061900/TraesCS4D03G0062000/TraesCS4D03G0062700/TraesCS4D03G0062800/TraesCS4D03G0062900/TraesCS4D03G0063000/TraesCS4D03G0063100* | response to gibberellin | 1.87E-18 | 5.10E-17 | 11 |
|  | *TraesCS4D03G0057400/TraesCS4D03G0061200/TraesCS4D03G0061300/TraesCS4D03G0061400/TraesCS4D03G0061500/TraesCS4D03G0061900/TraesCS4D03G0062000/TraesCS4D03G0062700/TraesCS4D03G0062800/TraesCS4D03G0062900/TraesCS4D03G0063000/TraesCS4D03G0063100* | leaf senescence | 4.06E-17 | 8.33E-16 | 12 |
|  | *TraesCS4D03G0061200/TraesCS4D03G0061300/TraesCS4D03G0061400/TraesCS4D03G0061500/TraesCS4D03G0061900/TraesCS4D03G0062000/TraesCS4D03G0062700/TraesCS4D03G0062900/TraesCS4D03G0063000/TraesCS4D03G0063100* | cysteine-type peptidase activity | 1.03E-16 | 4.94E-15 | 10 |
|  | *TraesCS4D03G0061200/TraesCS4D03G0061300/TraesCS4D03G0061400/TraesCS4D03G0061500/TraesCS4D03G0061900/TraesCS4D03G0062000/TraesCS4D03G0062700/TraesCS4D03G0062800/TraesCS4D03G0062900/TraesCS4D03G0063000/TraesCS4D03G0063100* | response to ethylene | 1.77E-15 | 2.91E-14 | 11 |
|  | *TraesCS4D03G0057900/TraesCS4D03G0058000* | IMP metabolic process | 1.24E-05 | 0.000145 | 2 |
|  | *TraesCS4D03G0057900/TraesCS4D03G0058000* | 'de novo' AMP biosynthetic process | 1.89E-05 | 0.000194 | 2 |
|  | *TraesCS4D03G0057900/TraesCS4D03G0058000* | adenylosuccinate synthase activity | 9.76E-06 | 0.000234 | 2 |
|  | *TraesCS4D03G0064200/TraesCS4D03G0064300* | beta-mannosidase activity | 4.72E-05 | 0.000756 | 2 |
|  | *TraesCS4D03G0064200/TraesCS4D03G0064300* | cellobiose glucosidase activity | 7.28E-05 | 0.000874 | 2 |
|  | *TraesCS4D03G0064200/TraesCS4D03G0064300* | beta-L-arabinosidase activity | 0.000131 | 0.00105 | 2 |
|  | *TraesCS4D03G0057400/TraesCS4D03G0060300* | sphingolipid biosynthetic process | 0.000416 | 0.00379 | 2 |
|  | *TraesCS4D03G0064200/TraesCS4D03G0064300* | beta-galactosidase activity | 0.000968 | 0.0053 | 2 |
|  | *TraesCS4D03G0058600/TraesCS4D03G0058900* | oxidoreductase activity, acting on single donors with incorporation of molecular oxygen, incorporation of two atoms of oxygen | 0.000993 | 0.0053 | 2 |
|  | *TraesCS4D03G0057800/TraesCS4D03G0064400* | preribosome, large subunit precursor | 0.000674 | 0.00741 | 2 |
|  | *TraesCS4D03G0058100* | NatB complex | 0.00292 | 0.0154 | 1 |
|  | *TraesCS4D03G0059900* | U2-type post-spliceosomal complex | 0.00292 | 0.0154 | 1 |
|  | *TraesCS4D03G0057800* | PeBoW complex | 0.0035 | 0.0154 | 1 |
|  | *TraesCS4D03G0064200/TraesCS4D03G0064300* | scopolin beta-glucosidase activity | 0.00434 | 0.0174 | 2 |
|  | *TraesCS4D03G0057700* | Cul4B-RING E3 ubiquitin ligase complex | 0.00582 | 0.0213 | 1 |
|  | *TraesCS4D03G0060300* | fatty acid elongation, saturated fatty acid | 0.00357 | 0.0244 | 1 |
|  | *TraesCS4D03G0060300* | fatty acid elongation, monounsaturated fatty acid | 0.00357 | 0.0244 | 1 |
|  | *TraesCS4D03G0060300* | fatty acid elongation, polyunsaturated fatty acid | 0.00357 | 0.0244 | 1 |
|  | *TraesCS4D03G0057800* | ribonucleoprotein complex binding | 0.00717 | 0.0246 | 1 |
|  | *TraesCS4D03G0058100* | peptide alpha-N-acetyltransferase activity | 0.00776 | 0.0248 | 1 |
|  | *TraesCS4D03G0059800* | nucleotidase activity | 0.00895 | 0.0269 | 1 |
|  | *TraesCS4D03G0060000* | ribosomal protein import into nucleus | 0.00536 | 0.0271 | 1 |
|  | *TraesCS4D03G0060000* | positive regulation of protein import into nucleus | 0.00536 | 0.0271 | 1 |
|  | *TraesCS4D03G0057700* | signal transduction involved in G2 DNA damage checkpoint | 0.00536 | 0.0271 | 1 |
|  | *TraesCS4D03G0059900* | generation of catalytic spliceosome for second transesterification step | 0.00595 | 0.0271 | 1 |
|  | *TraesCS4D03G0057400* | response to nutrient levels | 0.00595 | 0.0271 | 1 |
|  | *TraesCS4D03G0057400* | ceramide metabolic process | 0.00654 | 0.0282 | 1 |
|  | *TraesCS4D03G0064000* | translation release factor complex | 0.0093 | 0.0292 | 1 |
|  | *TraesCS4D03G0064600* | response to L-glutamate | 0.00713 | 0.0293 | 1 |
|  | *TraesCS4D03G0057400* | hydrolase activity, acting on carbon-nitrogen (but not peptide) bonds, in linear amides | 0.0119 | 0.0301 | 1 |
|  | *TraesCS4D03G0060800* | ethylene receptor activity | 0.0119 | 0.0301 | 1 |
|  | *TraesCS4D03G0060800* | ethylene binding | 0.0119 | 0.0301 | 1 |
|  | *TraesCS4D03G0059900* | nuclear retention of unspliced pre-mRNA at the site of transcription | 0.00832 | 0.0314 | 1 |
|  | *TraesCS4D03G0059800* | nucleotide catabolic process | 0.00891 | 0.0314 | 1 |
|  | *TraesCS4D03G0057400* | positive regulation of autophagy | 0.0101 | 0.0314 | 1 |
|  | *TraesCS4D03G0058100* | N-terminal peptidyl-methionine acetylation | 0.0101 | 0.0314 | 1 |
|  | *TraesCS4D03G0057700* | positive regulation of G2/M transition of mitotic cell cycle | 0.0107 | 0.0314 | 1 |
|  | *TraesCS4D03G0057700* | positive regulation of protein catabolic process | 0.0107 | 0.0314 | 1 |
|  | *TraesCS4D03G0064000* | cytoplasmic translational termination | 0.0113 | 0.0314 | 1 |
|  | *TraesCS4D03G0064600* | root system development | 0.0113 | 0.0314 | 1 |
|  | *TraesCS4D03G0059800* | pyrimidine nucleobase metabolic process | 0.0119 | 0.0314 | 1 |
|  | *TraesCS4D03G0057700* | protein monoubiquitination | 0.0119 | 0.0314 | 1 |
|  | *TraesCS4D03G0057400* | water homeostasis | 0.0119 | 0.0314 | 1 |
|  | *TraesCS4D03G0057800* | maturation of 5.8S rRNA from tricistronic rRNA transcript (SSU-rRNA, 5.8S rRNA, LSU-rRNA) | 0.0125 | 0.0319 | 1 |
|  | *TraesCS4D03G0064000* | translation release factor activity | 0.0137 | 0.0329 | 1 |
|  | *TraesCS4D03G0064000* | sequence-specific mRNA binding | 0.0155 | 0.0353 | 1 |
|  | *TraesCS4D03G0057700* | translesion synthesis | 0.0154 | 0.0383 | 1 |
|  | *TraesCS4D03G0064400* | ribosomal large subunit export from nucleus | 0.0172 | 0.0414 | 1 |
|  | *TraesCS4D03G0058600* | lipid oxidation | 0.0224 | 0.0472 | 1 |
|  | *TraesCS4D03G0064600* | MAPK cascade | 0.023 | 0.0472 | 1 |
|  | *TraesCS4D03G0064600* | root meristem growth | 0.023 | 0.0472 | 1 |
|  | *TraesCS4D03G0061100* | 1-phosphatidylinositol-4-phosphate 5-kinase activity | 0.0266 | 0.0491 | 1 |

^a^Gene ID (*Triticum aestivum*), T. *aestivum* gene transcripts and their domains that were available in Ensembl (using the transcript table link). ^b^FDR, False discovery rate, reflect the probability of false positive rate in the test.
